# Exosome-mediated cell-cell communication: a new perspective on the mechanisms and therapeutic potential of diabetic microvascular complications

**DOI:** 10.3389/fphar.2026.1839895

**Published:** 2026-05-29

**Authors:** Ye-xin Chen, Yi-shan Wu, Run-dong Yu, Yi-yu Dong, Jin-xi Zhao, Yao-fu Zhang

**Affiliations:** 1 Dongzhimen Hospital, Beijing University of Chinese Medicine, Beijing, China; 2 Beijing University of Chinese Medicine, Beijing, China; 3 Tsinghua University Yuquan Hospital, Beijing, China

**Keywords:** diabetic kidney disease, diabetic microvascular complications, diabetic peripheral neuropathy, diabetic retinopathy, exosomes

## Abstract

Diabetic microvascular complications, including diabetic kidney disease, diabetic retinopathy, and diabetic peripheral neuropathy, are associated with a growing burden and frequently present as comorbidities, posing substantial therapeutic challenges. Exosomes have been identified as key drivers in the pathogenesis of these conditions by mediating cell-cell communication. This review summarizes the biogenesis, cargo sorting, and uptake of exosomes, with emphasis on how these processes are reprogrammed under metabolic stress, converting exosomes from physiological regulators into carriers of pathological signals. A focused analysis is provided on how metabolic stress reshapes exosomal cargo profiles in each complication, leading to the enrichment of specific microRNAs, proteins, and lipids. These pathological exosomes establish aberrant communication networks among renal, retinal, and neural cells, through which inflammatory responses, oxidative stress, apoptosis, and fibrosis are amplified and vascular injury signals are transmitted, forming self-reinforcing pathological cycles. Exosomes also hold significant promise for clinical translation. Exosomes derived from body fluids carry molecules from injured cells and can serve as non-invasive biomarkers for early diagnosis. Exosome-based therapeutic strategies, particularly those involving stem cell-derived exosomes or exosomes modulated by antidiabetic drugs and natural products, offer multi-target approaches for microvascular intervention. Current challenges include elucidating cross-organ communication networks in comorbid conditions, advancing clinical standardization of exosomal biomarkers, and developing engineered exosomes for precision therapy. An exosome-mediated cell-cell communication perspective provides a more integrated framework for understanding diabetic microvascular comorbidities and may inform the development of multi-complication co-targeting strategies.

## 1 Introduction

Diabetes has become a major global public health challenge, with approximately 589 million individuals affected worldwide, and its prevalence continues to rise (Genitsaridi et al., 2026). The chronic hyperglycemia associated with diabetes persistently damages the systemic microvascular network, leading to a series of severe complications. Among these, diabetic kidney disease (DKD), diabetic retinopathy (DR), and diabetic peripheral neuropathy (DPN) are the most common and representative microvascular complications (Singhal et al., 2025). They are the leading causes of kidney failure, vision loss, and foot ulcers or amputations in diabetic patients, significantly reducing their quality of life and imposing a heavy socio-economic burden (Zhang L. et al., 2025; Purola et al., 2022; Hicks and Selvin, 2019). Epidemiological studies have revealed that the prevalence of DKD, DR, and DPN in diabetic populations exceeds 20% (Zhang L. et al., 2025). Moreover, these complications often do not occur in isolation in clinical settings. Rather, they coexist within the same patient and interact with one another, resulting in a complex comorbid condition that complicates treatment strategies (Satapathy et al., 2025). Traditional therapies targeting single complications are often limited in their effectiveness when faced with progressive microvascular damage affecting multiple systems (LeRoith et al., 2019). This highlights the urgent need to explore common pathophysiological mechanisms and intervention targets from a more fundamental perspective.

In recent years, extracellular vesicles (EVs), particularly exosomes, have garnered widespread attention as key mediators of cell-cell communication in diabetes and its complications. Exosomes are nanoscale lipid bilayer vesicles actively released by cells, carrying a variety of bioactive molecules, including proteins, nucleic acids, and lipids. Under normal physiological conditions, they facilitate cell-cell signaling and contribute to the maintenance of tissue homeostasis (Chen YF. et al., 2024). However, under prolonged conditions of hyperglycemia, inflammation, and metabolic stress, the biogenesis, cargo selection, and delivery processes of exosomes undergo pathological alterations (Su et al., 2025). These exosomes transition from homeostasis regulators to carriers of damaging signals, forming abnormal cell-cell communication networks that disseminate and amplify local metabolic disturbances. This process cooperatively promotes microvascular injury, inflammatory responses, cell death, and fibrosis in multiple target organs, including the kidneys, retina, and nerves (Jia et al., 2018; Lv et al., 2020; Liu Y. et al., 2022; Wu et al., 2016; Tsai et al., 2023).

Therefore, a deeper understanding of the role of exosome-mediated cell-cell communication in the onset and progression of diabetic microvascular complications, particularly by elucidating the common mechanisms between different complications, not only provides a new perspective for understanding the nature of the disease but also provides a theoretical foundation for developing multi-target therapeutic strategies that can intervene in multiple comorbidities. This review focuses on the key mechanisms underlying exosome-mediated cell-cell communication in diabetic microvascular complications and their comorbid states, and explores its potential applications in early diagnosis and clinical translation.

## 2 Biogenesis and uptake of exosomes

Exosomes are a type of EVs secreted by various cell types, typically ranging in size from 30 to 100 nm in diameter. They play a critical role in cell-cell communication by delivering biomolecules, such as nucleic acids, proteins, and lipids, to recipient cells. Exosomes are enriched with a variety of proteins, including heat shock proteins, tetraspanins, Endosomal Sorting Complex Required for Transport (ESCRT)-associated proteins, cytoskeletal proteins, and GTPases. These molecules are involved not only in the formation, cargo sorting, and release of exosomes but also in antigen presentation, membrane microdomain organization, endosomal system regulation, and the maintenance of cytoskeletal structure and function (Khan et al., 2025).

**FIGURE 1:**
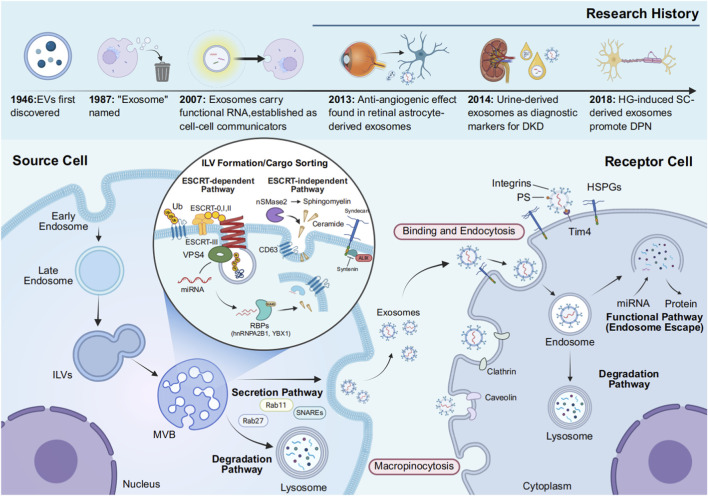
This schematic illustrates the molecular pathway of exosomes, from biogenesis and secretion to uptake and function in recipient cells. Exosomes arise from the endosomal pathway, where cargo is sorted via ESCRT-dependent or -independent mechanisms before being secreted extracellularly under the regulation of related proteins. Recipient cells internalize exosomes through multiple ways including endocytosis or macropinocytosis. Internal contents can either escape endosomes to activate functional pathways or undergo lysosomal degradation. The timeline traces exosome research from foundational discovery to their roles in diabetic microvascular complications, offering a clear framework for understanding their pathophysiological roles in disease. DKD, Diabetic Kidney Disease; HG, High Glucose; SCs, Schwann Cells; DPN, Diabetic Peripheral Neuropathy; ILVs, Intraluminal Vesicles; MVBs, Multivesicular Bodies; ESCRT, Endosomal Sorting Complex Required for Transport; VPS4, Vacuolar Protein Sorting 4; RBPs, RNA-binding proteins; PS, Phosphatidylserine; HSPGs, Heparan Sulfate Proteoglycans.

EVs were first reported in 1946 (Xu G. et al., 2025). In 1987, Johnstone et al. formally named these vesicles released by multivesicular bodies as “exosomes,” proposing their primary function as cellular waste disposal (Johnstone et al., 1987). In 2007, Valadi et al. discovered that exosomes contain functional mRNA and microRNA (miRNA), which can be transferred to recipient cells to exert biological effects (Valadi et al., 2007). It established exosomes as important mediators of cell-cell communication. By the 2010s, the role of exosome-mediated cell-cell communication in diabetic microvascular complications began to receive increased attention (Jia et al., 2018). In 2013, a study reported that exosomes derived from retinal astrocytes contain anti-angiogenic components (Hajrasouliha et al., 2013). In 2014, the analysis of miRNA and protein compositions in urinary-derived exosomes from patients with DKD provided evidence supporting the use of urinary-derived exosomes as biomarkers (Zubiri et al., 2014). In 2018, research showed that exosomes released by Schwann Cells (SCs) under high glucose (HG) conditions can promote the progression of DPN (Jia et al., 2018). Since then, more studies have focused on the role of exosome-mediated communication in the pathogenesis of diabetic microvascular complications. A deeper understanding of the mechanisms involved in exosome biogenesis, cargo loading, and uptake is essential to comprehending how they mediate pathological cell communication under conditions of hyperglycemia, inflammation, and metabolic stress, ultimately leading to microvascular injury (Figure 1).

### 2.1 Molecular pathways of exosome formation

The classical biogenesis of exosomes begins with the endocytic pathway. Early endosomes are formed by the invagination of the plasma membrane, and upon maturation into late endosomes, their limiting membrane buds inward to generate Intraluminal Vesicles (ILVs). Structures enriched with ILVs are referred to as multivesicular bodies (MVBs). The fate of the MVB determines whether exosomes are released. MVBs can either fuse with lysosomes, leading to the degradation of ILVs, or fuse with the plasma membrane, releasing exosomes into the extracellular space (Johnstone et al., 1987; Théry et al., 2002; Raposo and Stoorvogel, 2013; van Niel et al., 2018; Pegtel and Gould, 2019; Pan et al., 1985). This balance between secretion and degradation is dynamically regulated by various mechanisms, including the ESCRT complex and lipid metabolism (van Niel et al., 2018; Pegtel and Gould, 2019; Ostrowski et al., 2010; Savina et al., 2002; Savina et al., 2005; Guo et al., 2017; Yanagawa et al., 2024). The ESCRT-dependent pathway serves as the core framework for ILV formation and cargo sorting. ESCRT-0, -I, -II, -III complexes, along with auxiliary proteins like Vacuolar Protein Sorting 4 (VPS4), cooperate to recognize ubiquitinated membrane proteins, driving membrane deformation, neck scission, and membrane fission to promote ILV generation (Wollert and Hurley, 2010; Colombo et al., 2013; Baietti et al., 2012; Jackson et al., 2017). Functional studies have shown that inhibition of VPS4 can broadly affect the release of exosomes, highlighting the essential role of the ESCRT mechanism in this process (Jackson et al., 2017). The ESCRT-independent pathway emphasizes the role of membrane lipid microdomains. For example, neutral sphingomyelinase 2 (nSMase2) hydrolyzes sphingomyelin to generate ceramide, whose inherent conical structure induces local membrane curvature and promotes ILV budding (Colombo et al., 2013; Trajkovic et al., 2008). Notably, nSMase2 activity can also regulate the acidification state of the MVB lumen by affecting the assembly of the V-ATPase complex, eventually influencing the decision between MVB secretion or degradation (Choezom and Gross, 2022). This mechanism links lipid metabolism with organelle function. Additionally, tetraspanins like CD63 participate in the sorting of cargo into ILVs by organizing tissue-specific membrane microdomains, illustrating their role as organizing centers for secretory MVBs (Colombo et al., 2013). Beyond these core mechanisms, specific protein networks, such as the syndecan/syntenin/ALIX axis, finely regulate the entry of specific cargo into exosomes by connecting cell membrane receptors with ESCRT-associated component (Baietti et al., 2012; Ghossoub et al., 2014). The transport, anchoring, and fusion of MVBs to the plasma membrane are primarily mediated by the Rab family of small GTPases (e.g., Rab27a/b, Rab11) and Soluble NSF Attachment Protein Receptor (SNARE) proteins, determining the final efficiency of exosome release (Ostrowski et al., 2010; Savina et al., 2002; Savina et al., 2005).

Metabolic stress acts directly on exosome biogenesis and dynamically regulates ILV formation, MVB fate determination, and secretory efficiency. At the level of the ESCRT-dependent pathway, hypoxia provides a representative example. It can promote exosome biogenesis and cargo sorting through the HIF-1α/HRS axis, directly influencing ESCRT-mediated sorting (Wang Y. et al., 2025). With respect to MVB fate, hypoxia can downregulate ATP6V1A via HIF-1α, disrupt lysosomal acidification, and inhibit MVB trafficking toward the degradative pathway (Wang X. et al., 2023). In the lipid-driven ESCRT-independent pathway, palmitate-induced lipotoxicity has been shown to promote the release of exosome enriched in C16:0 ceramide through an IRE1α-dependent mechanism. This finding indicates that metabolic stress can directly drive membrane invagination and vesicle budding through the sphingomyelin/ceramide axis (Kakazu et al., 2016). At the final exocytic stage, glycolytic reprogramming can promote exosome secretion by upregulating PKM2, which induces SNAP23 phosphorylation and strengthens SNARE complex-mediated membrane fusion (Wei et al., 2017). Notably, HG may suppress exosome secretion by promoting FOXO1 phosphorylation and inactivation, thereby downregulating RAB27B (Zeng et al., 2021). In contrast, proteinuric stress or albumin overload stress can enhance exosome release through the IRF-1/Rab27a axis (Feng et al., 2020). Taken together, metabolic stress dynamically reprograms ESCRT-mediated sorting, lipid-driven budding, Rab-dependent trafficking, depending on the type and duration of stress as well as the cellular context. These changes provide a mechanistic basis for the establishment of subsequent pathological communication networks.

### 2.2 Sorting mechanisms and loading processes of exosome cargo

The function of exosomes is highly dependent on the cargo they carry. These cargoes are not randomly packaged but are selectively sorted through active sorting mechanisms, with distinct characteristics at different molecular levels, including proteins, nucleic acids, and lipids. The sorting of protein cargo is primarily governed by signal recognition mechanisms. The classical ESCRT pathway sorts membrane proteins by recognizing ubiquitin tag (Wollert and Hurley, 2010; Colombo et al., 2013; Baietti et al., 2012). Ubiquitination of the cytoplasmic tail of transmembrane proteins can be recognized and aggregated by ESCRT-0/ESCRT-I, which then directs the proteins to the ESCRT-III-mediated membrane neck scission process, coupling cargo sorting with ILV formation (Wollert and Hurley, 2010). Additionally, membrane microdomains formed by tetraspanins provide sorting platforms for specific protein groups (Colombo et al., 2013). Changes in the metabolic environment can directly regulate the sorting process. For example, cholesterol homeostasis can affect MVB properties via caveolin-1, reshaping the protein composition of exosomes (Yanagawa et al., 2024). Lipid overload in diabetic conditions may disrupt the selective loading of protein cargo by altering membrane lipid composition and endosomal homeostasis (Jackson et al., 2017; Cunha et al., 2024). The sorting of nucleic acid cargo, particularly miRNAs, is more complex and involves sequence-specific recognition processes. Several RNA-binding proteins (RBPs) play key roles in this process. hnRNPA2B1 promotes miRNA incorporation into exosomes by recognizing specific sequence motifs and being modified by SUMOylation (Villarroya-Beltri et al., 2013). SYNCRIP (hnRNPQ) is responsible for the sorting of specific miRNAs in hepatocytes (Santangelo et al., 2016). The well-studied YBX1 protein can recognize specific miRNA sequences and form condensates that serve as efficient selective sorting platforms through liquid-liquid phase separation (LLPS) (Liu et al., 2021). In addition to protein-mediated recognition mechanisms, the sequence characteristics of miRNAs themselves also determine their sorting fate. Research by Garcia-Martin et al. revealed that certain guanine-cytosine-rich motifs promote the sorting of miRNAs into exosomes, while other motifs tend to retain them within the cell (Cunha et al., 2024). This mechanism provides new insights into the tissue origin and disease-specific changes of miRNAs in circulating exosomes. Lipid cargoes are not only structural components of the exosome membrane but also important bioactive signaling molecules. For instance, ceramide can drive ILV budding and itself acts as a pro-apoptotic or pro-inflammatory signal that is packaged into exosomes for transmission (Trajkovic et al., 2008; Zietzer et al., 2021). It is important to emphasize that the cargo composition of exosomes exhibits high heterogeneity. High-resolution separation techniques have revealed that even distinct EV subpopulations derived from the same cell may carry vastly different protein and RNA components, triggering varying responses in recipient cells (Huang et al., 2022).

### 2.3 Exosome release and uptake

Exosome-mediated cell-cell communication ultimately relies on the recognition, internalization, and functional delivery of cargo to recipient cells. This process begins with the specific recognition of exosomes by the target cell surface. The identity markers on the exosomal membrane, such as specific integrin profiles, phosphatidylserine (PS), and glycosaminoglycan-binding proteins, determine their tissue tropism and cellular targeting. For instance, the specific integrin combinations on tumor-derived exosomes dictate their preferential uptake by lung or liver cells. This homing mechanism provides important insights into the specific damage observed in different microvascular beds in diabetes (Hoshino et al., 2015). On the recipient cell surface, heparan sulfate proteoglycans (HSPGs) and PS receptors such as Tim4 have also been identified as key molecules mediating exosome internalization (Christianson et al., 2013; Miyanishi et al., 2007).

The internalization pathways of exosomes are diverse and primarily include clathrin-mediated endocytosis, caveolin-dependent endocytosis, macropinocytosis, and phagocytosis (Mulcahy et al., 2014; Mathieu et al., 2019). Different exosome subpopulations may preferentially use specific endocytic pathways, while a single cell may simultaneously employ multiple uptake mechanisms (Mulcahy et al., 2014; Mathieu et al., 2019). Once internalized, exosomes typically enter the endosomal-lysosomal pathway, where the risk of cargo degradation exists. Therefore, the ability to achieve functional delivery depends on whether the cargo can be effectively released or escape from the endosomal system (Théry et al., 2002; Zhuo et al., 2024). Imaging studies have shown that only a subset of exosomes can release their cargo, such as miRNAs or proteins, into the cytoplasm of recipient cells through membrane fusion or by disrupting the endosomal membrane before the acidification and degradation of the endosome (Théry et al., 2002). Engineering studies have revealed that increasing the cholesterol content in exosomal membranes significantly enhances their fusion efficiency with the cell membrane, bypassing the endosomal degradation pathway and enabling the direct delivery of large molecules such as siRNA into the cytoplasm (Zhuo et al., 2024). This indicates that the lipid composition of the exosomal membrane is a key variable determining the uptake and delivery efficiency.

In summary, the biogenesis, selective loading, and targeted uptake of exosomes form a highly regulated cycle of cell-cell communication. Under the context of metabolic disorders in diabetes, hyperglycemia, lipotoxicity, and inflammatory signals can reprogram multiple steps of this cycle, transforming exosomes from homeostasis regulators to promoters of microvascular injury.

## 3 Common mechanisms of exosome-mediated cell-cell communication in diabetic microvascular complications

Although DKD, DR, and DPN affect different organs, their pathological progression revolves around the structural and functional damage of microvasculature. Persistent hyperglycemia and metabolic stress drive microvascular barrier disruption, abnormal blood flow perfusion, and organ dysfunction through multiple pathological processes, including oxidative stress, inflammation, cell apoptosis, and fibrosis. Recent studies have revealed that exosome-mediated cell-cell communication plays a central role in transmitting and amplifying these pathological events. Exosomes, reprogrammed by metabolic stress, establish abnormal cell-cell communication networks by carrying specific miRNAs, proteins, and lipid signaling molecules. These exosomes spread local metabolic damage signals to adjacent and even distant target cells, eventually amplifying the inflammatory response, exacerbating barrier disruption, and promoting cell death and fibrosis.

### 3.1 Metabolic stress influences exosome biogenesis and their selective uptake in different target organs

Metabolic stress signals influence the biogenesis of exosomes. HG activates nSMase2, promoting the generation of exosomes enriched with ceramide. These vesicles facilitate the transfer of lipotoxic signals between epithelial cells (ECs), inducing cell apoptosis (Zietzer et al., 2021). In pancreatic β-cells, inflammation also induces the release of a specific exosome subpopulation enriched with ceramide through an nSMase2-dependent mechanism (Xu J. et al., 2025). Additionally, the generation of membrane ceramide by CD36 of EC has been found to be associated with exosomal fatty acid transfer and circulating lipid levels (Peche et al., 2023). More importantly, metabolic dysregulation profoundly alters the types of cargo carried by exosomes. A greater load of pathogenic proteins and miRNAs is incorporated into exosomes, enhancing the transmission of pathological signals. Several studies have confirmed that exosomes derived from renal tubular epithelial cells (TECs), glomerular endothelial cells (GECs), retinal pigment epithelial cells (RPEs), and SCs are enriched in pro-inflammatory miRNAs (Jia et al., 2018; Lv et al., 2020; Wu et al., 2017; Zhang et al., 2019). At the same time, these exosomes carry multiple pro-apoptotic and pro-fibrotic signals, collectively driving tissue damage progression across multiple organ (Jia et al., 2018; Tsai et al., 2023; Qiu et al., 2025).

Whether target cells can effectively receive exosome signals is crucial for determining whether exosome-mediated cell-cell communication can promote microvascular damage. The affinity of exosomes for specific target cells is determined by molecules such as integrins, PS, and glycosaminoglycan-binding proteins present on the exosomal surface. Different cells, due to their distinct receptor profiles and endocytic pathways, exhibit varying preferences for the uptake of exosome subpopulations. In the renal, GECs and podocytes are enriched with glycosaminoglycans and HSPGs on their surfaces, making them more susceptible to the efficient internalization of locally derived exosomes (Matsumoto et al., 2017). In contrast, interstitial macrophages, with their stronger phagocytic ability, are more likely to internalize exosomes with exposed PS or specific integrin/tetraspanin features (Hoshino et al., 2015; Matsumoto et al., 2017). In retinal endothelial cells (RECs), exosomes dock through surface HSPGs and integrins, followed by internalization via clathrin- or caveolin-mediated endocytosis, making them more likely to directly affect tight junctions and barrier permeability (Hoshino et al., 2015; Mulcahy et al., 2014; Gad et al., 2024). Microglial cells and infiltrating macrophages preferentially internalize exosomes through phagocytosis or macropinocytosis, regulated by PS recognition receptors and scavenger receptors, leading to the accumulation of inflammatory exosomes and the formation of sustained amplified immune responses in the local area (Matsumoto et al., 2017; Beltramo et al., 2023). Neurons and their axonal terminals can internalize exosomes via synaptic-related endocytosis, followed by retrograde transport along microtubules to the cell body (Ren et al., 2019; Frühbeis et al., 2013). In the peripheral nervous system, exosomes from SCs are typically internalized in regions with active membrane renewal, such as the growth cone and Ranvier’s node, mainly through clathrin-dependent endocytosis or macropinocytosis at the axonal terminals. These exosomes can undergo retrograde transport along the axon, allowing signals from glial cells to directly reach the dorsal root ganglion (DRG) neurons’ cell bodies. ECs of the neurovascular unit primarily rely on caveolin-associated endocytosis and HSPG-mediated adhesion for exosome uptake. Under inflammatory conditions, the upregulation of adhesion molecules further enhances exosome docking and transendothelial transport, promoting the accumulation of abnormal signals near the blood-nerve barrier (Ren et al., 2019; Lopez-Verrilli et al., 2013).

### 3.2 Exosome-mediated cell-cell communication amplifies pathological signals of metabolic stress

Chronic low-grade inflammation induced by hyperglycemia activates the NF-κB signaling pathway, promotes the release of pro-inflammatory cytokines, enhances oxidative stress, and disrupts endothelial nitric oxide synthase, directly damaging vascular ECs. This process represents the core mechanism underlying diabetic microvascular complications. Exosome-mediated cell-cell communication amplifies these initial inflammatory signals systemically, accelerating the progression of microvascular damage (Grzybowska-Adamowicz and Zmysłowska, 2026; Atak Tel et al., 2025).

Exosome-mediated cell-cell communication amplifies inflammatory signals in the diabetic microenvironment. At the level of signal transmission, exosomes achieve transcellular derepression of inflammatory pathways through the delivery of miRNAs. Under hyperglycemic stress, exosomes enriched with specific miRNAs are released by various source cells, such as TECs and fibroblasts, and are internalized by target cells, including macrophages and neuronal cells. The miRNAs carried by these exosomes target and suppress key negative regulators of the NF-κB pathway, including the SOCS family, consequently relieving the inhibition of inflammatory signals and triggering a downstream cytokine storm (Lv et al., 2020; Wu et al., 2021). At the level of signal recognition, exosomes, as directly recognizable signaling entities, activate pattern recognition receptors through membrane proteins or ligands they carry, directly initiating inflammatory pathways at the upstream level. Exosomes can trigger the activation of TLR4 on target cells, including RECs and podocytes. This activation occurs through interactions involving exosomal membrane proteins or the delivery of inflammatory mediators carried by the exosomes (Zhang et al., 2019; Wang Z. et al., 2022). At the signal integration level, exosomes simultaneously influence various oxidative stress and inflammatory pathways, promoting the formation of positive feedback loops. For example, fibroblast-derived exosomes exacerbate local inflammation by inhibiting autophagy, promoting macrophage polarization, and activating the NLRP3 inflammasome and oxidative stress pathways (Xu Z. et al., 2025).

Macrophage polarization is also a key mechanism by which exosome-mediated cell-cell communication transforms inflammatory signals into a sustained immune microenvironment disturbance. On one hand, several studies have shown that exosomes derived from TECs and Müller cells are enriched with various pro-inflammatory miRNAs. These exosomes promote M1 macrophage polarization by targeting key inhibitory genes that maintain macrophage homeostasis (Lv et al., 2020; Xu Z. et al., 2025). On the other hand, exosomes can directly deliver protein signals or epigenetic modifying enzymes, determining macrophage fate at the levels of membrane receptor activation and transcriptomic reprogramming. For example, after macrophages internalize exosomes, their surface Notch receptors can be directly activated by ligands such as Dll4 present on the exosomal membrane, which then transcriptionally regulate M1 polarization gene programs (Liu JL. et al., 2023). Exosomes from M1 macrophages can also exacerbate local inflammation and apoptosis by modifying the methylation levels of specific mRNAs (Li Y. et al., 2024). Moreover, exosomes released by M1-polarized macrophages can directly attack ECs and form a self-amplifying damage cycle. In DKD, miR-21a-5p in exosomes from M1 macrophages can induce podocyte apoptosis (Liang et al., 2022). In DR, exosomes derived from M1 microglia, containing proteins such as CCL2, MMP2/9, and miR-21, lead to blood-retinal barrier (BRB) disruption and pathological angiogenesis (Beltramo et al., 2023). In summary, as key mediators of cell-cell communication, exosomes systematically transmit and amplify inflammatory signals, establishing a self-reinforcing inflammatory cycle that greatly accelerates the progression from localized injury to widespread tissue pathology. These shared core mechanisms of inflammation signal amplification across different microvascular beds also provide insights into early interventions for diabetic microvascular comorbidities (Table 1).

**TABLE 1:** Exosome-mediated cell-cell communication amplifies pathological signals of metabolic stress.

Disease background	Species	Exosomal cargo	Exosome source	Target cells	Mechanism	Functional outcome	References
DKD	Mouse	LRG1, TRAIL	TECs and Macrophages from HFD-induced T2DM mice	Macrophages; TECs	Lipotoxic TEC-derived exosomes enriched with LRG1 activate macrophages via TGFβ-R1; activated macrophage-derived exosomes enriched with TRAIL induce apoptosis in injured TECs via DR5	Amplifies tubulointerstitial inflammation and epithelial cell apoptosis, propagating initial lipotoxic stress	Jiang et al. (2022)
DKD	Mouse	Dll4	HG-induced TECs (HK-2)	Macrophages (THP-1)	Epsin1 mediates exosomal sorting of Dll4, activating macrophage Notch1 signaling and promoting M1 polarization	Exacerbates inflammatory stress by activating macrophages, leading to tubulointerstitial damage	Liu et al. (2023a)
Kidney injury	Mouse	miR-19b-3p	TECs from LPS-induced AKI mouse model and ADR-induced chronic proteinuric kidney disease model	Macrophages	Exosomal miR-19b-3p targets SOCS-1 to activate NF-κB, inducing M1 macrophage polarization	Amplifies inflammatory cascade through sustained M1 macrophage activation	Lv et al. (2020)
DKD	Mouse	miRNAs (e.g., miR-193a-3p, miR-1260B, miR-3175)	HG-induced macrophages (RAW264.7)	—	ERK regulates macrophage polarization via NF-κB/JAK-STAT, altering exosomal miRNA profile to promote M1 phenotype	Propagates pro-inflammatory signaling by reprogramming macrophage exosomal cargo	Shen et al. (2021)
DKD	Human	miR-221	HG-induced Podocytes	PTECs	Exosomal miR-221 targets DKK2 to activate Wnt/β-catenin signaling, inducing PTEC dedifferentiation	Transduces stress signals from podocytes to tubular cells, expanding injury to the tubulointerstitium	Su et al. (2020)
DKD	Mouse	miR-92a-1-5p	HG-induced PTECs	MCs	Exosomal miR-92a-1-5p targets reticulocalbin-3 in MCs, inducing ER stress and myofibroblast transdifferentiation	Transmits ER stress signals to mesangial cells, promoting glomerular fibrosis	Tsai et al. (2023)
DKD	Human	METTL14	M1 macrophages	GECs	Exosomal METTL14 mediates m6A modification of PAQR3 in GECs, promoting apoptosis, inflammation, and oxidative stress	Amplifies oxidative and inflammatory stress in GECs	Li et al. (2024a)
DKD	Mouse	miR-21-5p	HG-induced macrophages (RAW264.7)	Podocytes	Exosomal miR-21-5p targets the 3′-UTR of A20 mRNA, inhibiting A20 expression, which leads to NLRP3 inflammasome activation, ROS production, and podocyte pyroptosis	Amplifies podocyte inflammatory injury and pyroptosis, propagating glomerular damage	Ding et al. (2021)
DR	Mouse	lncRNA OGRU	HG-induced Müller cells	Microglia	Exosomal OGRU acts as a ceRNA sponge for miR-320-3p, miR-221-3p, and miR-574-5p, derepressing their respective targets AR, PFKFB3, and GLUT1, promoting M1 microglia polarization	Amplifies neuroinflammation by inducing microglial M1 polarization	Fu et al. (2024)
DR	Human	Claudin-5 and other proteins	Vitreous from T2DM Patients	Macrophages	Vitreous exosomes deliver upregulated Cldn5 to macrophages, upregulating TNFα and IL-1β mRNA expression	Propagates inflammatory signaling to infiltrating macrophages	Shan et al. (2024)
DR	Human	Inflammasome-related cargo (NLRP3, IL-1β, etc.)	DR-like conditions-induced ARPE-19 cells	—	Exosomes carry increased inflammasome components, activating NLRP3 inflammasome in RECs	Amplifies inflammatory cascade via transmission of inflammasome signals	Louie et al. (2025)
DR	Rat	CXCL10	Platelet-rich plasma from diabetic rats	RECs	Exosomal CXCL10 activates TLR4 pathway in RECs, inducing oxidative stress and inflammation	Amplifies endothelial injury and inflammatory stress, compromising retinal vascular integrity	Zhang et al. (2019)
DPN	Mouse	Proteins and miRNAs (e.g., involved in axon guidance)	Keratinocytes from HFD mice	DRG neurons	HFD alters keratinocyte-derived exosomal cargo, dysregulating skin-nerve signaling	Propagates metabolic stress signals from periphery to sensory neurons, contributing to axonal degeneration	Coy-Dibley et al. (2024)
DPN	Rat	miR-221	Serum exosomes from DPN rats	Neuronal cells (SH-SY5Y)	Exosomal miR-221 targets SOCS3, derepressing NF-κB and downstream inflammatory factors (PEG2, BK, IL-6, IL-1β, TNF-α)	Amplifies neuroinflammation and pain signaling	Wu et al. (2021)
DPN	Rat	miR-21	HG-induced SCs	DRG neurons	HG downregulates miR-21 in SC-derived exosomes, impairing AKT signaling-mediated neurite outgrowth	Propagates metabolic stress by transferring reduced regenerative signals, inhibiting neuronal repair	Liu et al. (2022b)
DPN	Human	miR-28, miR-31a, miR-130a	HG-induced SCs	DRG neuron axons	Exosomes transfer these miRs to axons, downregulating target proteins (DNMT-3a, NUMB, SNAP25, GAP-43) required for axon growth	Amplifies peripheral neuropathy by transmitting stress-induced inhibitory signals to neurons	Jia et al. (2018)
Diabetic endothelial dysfunction	Human	miR-22-5p	Macrophages treated with AGE	HUVECs	Exosomal miR-22-5p targets FOXP1 in ECs, activating inflammatory pathways (VCAM-1, ICAM-1, MCP-1) and suppressing eNOS phosphorylation	Amplifies endothelial dysfunction by propagating inflammatory signals, impairing proliferation, migration, and tube formation	Ji et al. (2025)

DKD, diabetic kidney disease; LRG1, Leucine-rich α-2 glycoprotein 1; TRAIL, Tumor Necrosis Factor-related Apoptosis-inducing Ligand; TECs, Renal Tubular Epithelial Cells; HFD, High-Fat Diet; T2DM, Type 2 Diabetes Mellitus; Dll4, Delta-like Ligand 4; HG, high glucose; LPS, lipopolysaccharide; AKI, acute kidney injury; SOCS, suppressors of cytokine signaling; miRNAs, microRNA; ERK, Extracellular Signal-Regulated Kinase; DKK2, Dickkopf-related protein 2; MCs, Mesangial Cells; METTL14, Methyltransferase-like protein 14; GECs, Glomerular Endothelial Cells; lncRNA, long non-coding RNA; ceRNA, Competing Endogenous RNA; AR, aldose reductase; GLUT1, Glucose Transporter 1; RECs, Retinal Endothelial Cells; CXCL10, C-X-C Motif Chemokine Ligand 10; DPN, diabetic peripheral neuropathy; SCs, Schwann Cells; DRG, dorsal root ganglion; NUMB, NUMB, endocytic adaptor protein; SNAP25, Synaptosomal-Associated Protein 25; AGE, advanced glycation end products; HUVECs, Human Umbilical Vein Endothelial Cells; FOXP1, Forkhead Box Protein P1; ECs, Epithelial Cells; VCAM-1, Vascular Cell Adhesion Molecule 1; ICAM-1, Intercellular Adhesion Molecule 1; eNOS, endothelial nitric oxide synthase.

### 3.3 Exosome-mediated cell-cell communication disrupts microvascular homeostasis and promotes structural damage

Exosome-mediated cell-cell communication amplifies inflammatory signals, creating a microenvironment conducive to vascular damage. At the same time, as carriers of various bioactive molecules, exosomes directly affect microvascular functional cells, interfering with homeostatic regulatory networks and compromising barrier integrity, ultimately driving irreversible damage such as cell apoptosis and tissue fibrosis.

As carriers of non-coding RNAs, exosomes systematically disrupt the vascular homeostatic regulatory network. Upon entering target cells, the miRNAs and long non-coding RNA (lncRNAs) they carry regulate the activity of key pathways, including Notch, Vascular Endothelial Growth Factor (VEGF), and Wnt/β-catenin. In DKD, exosomes derived from GECs deliver miR-30a-5p to target the Notch1/VEGF signaling axis, interfering with endothelial function in kidney microvasculature (Ning et al., 2024). In DR, exosomes from Müller cells deliver miR-92a-3p, which suppresses Notch-1 receptors on retinal ganglion cells (RGCs) (Qiu et al., 2025). During the proliferative stage of DR, vitreous-derived exosomes activate the Wnt/β-catenin pathway via lncRNA, promoting fibrous membrane formation (Hu et al., 2023). These events disrupt the microvascular self-regulation at the signaling pathway level, laying the foundation for structural damage.

Simultaneously, exosomes directly initiate endothelial damage and barrier dysfunction by delivering effector proteins. Exosomes released by ECs under HG conditions directly carry TGF-β1 protein and its mRNA. Upon internalization by MCs or podocytes, they activate the TGF-β1/Smad3 fibrosis pathway, leading to perivascular matrix proliferation and disruption of the filtration barrier (Wu et al., 2016; Wu et al., 2017). Other studies have shown that exosomes enriched with IgG from diabetic plasma can activate the complement classical pathway independent of cell uptake, leading to the deposition of membrane attack complexes on RECs, resulting in direct cytolytic effects and BRB breakdown (Huang et al., 2020; Huang et al., 2018). The activated microglia-derived exosomes can degrade the extracellular matrix and chemotactic immune cells, exacerbating local retinal damage (Beltramo et al., 2023). The direct effects of these effector proteins physically disrupt endothelial junctions and barrier integrity.

As functional disruption and structural damage accumulate, exosomes ultimately drive the occurrence of terminal outcomes, such as cell apoptosis and tissue fibrosis. Under HG conditions, damaged cells propagate apoptotic and fibrotic signals to neighboring cells via exosomal cargo such as miRNAs and proteins, exemplified by GECs which undergo autocrine-induced apoptosis mediated by exosomal miR-30a-5p (Ning et al., 2024). In DR, exosome-mediated miR-483-5p/IGF-1R (Cao et al., 2024) and miR-18b/NF-κB (Xu et al., 2021) axes are directly associated with retinal cell apoptosis. Pro-fibrotic signals are similarly pivotal. In DKD, exosomes derived from TECs upregulate SMAD2 through the circ_0008529/miR-185-5p axis, directly driving fibrosis (Niu et al., 2023). During the proliferative stage of DR, vitreous-derived exosomes activate the Wnt/β-catenin pathway through lncRNA, promoting fibrous membrane formation (Hu et al., 2023). Exosomes released by damaged parenchymal cells, enriched with regulatory non-coding RNAs and effector proteins targeting key nodes in apoptosis and fibrosis pathways, are internalized by neighboring microvascular functional cells or neurons. By upregulating pro-fibrotic signals or inhibiting pro-survival kinase activity, these exosomes ultimately induce apoptosis, mesenchymal transition, and abnormal extracellular matrix remodeling in target cells (Wu et al., 2016; Gu et al., 2020; Liu YP. et al., 2022) (Table 2)

**TABLE 2:** Exosome-mediated cell-cell communication disrupts microvascular homeostasis and promotes structural damage.

Disease background	Species	Exosomal cargo	Exosome source	Target cells	Mechanism	Functional outcome	References
DKD	Mouse	miR-15b-5p	HG-induced MCs	mouse MCs	Exosomal miR-15b-5p targets Bcl-2, inducing MC apoptosis	Propagates apoptotic signals within mesangium, contributing to glomerulosclerosis and renal function decline	Tsai et al. (2020)
DKD	Mouse	Proteins (22 differentially expressed proteins, e.g., Eno1, Hspa8, Txn1, etc.)	HG-induced tubular cells (BUMPT cells)	Fibroblasts (NRK-49F)	HG alters tubular exosomal protein cargo; exosomes deliver these proteins to fibroblasts, inducing proliferation and activation	Transmits fibrotic signals from injured tubules to interstitium, driving renal interstitial fibrosis	Wen et al. (2020)
DKD	Mouse	miR-30a-5p	HG-induced GECs	GECs	miR-30a-5p is downregulated in HG-induced GECs and their exosomes, which targets Notch1 3′-UTR and downregulates Notch1/VEGF signaling	Exacerbates EMT and abnormal angiogenesis and contributes to renal dysfunction	Ning et al. (2024)
DKD	Human	hsa_circ_0006382, hsa_circ_0019539	Serum from DKD patients	TECs (NRK-52E)	Exosomal circRNAs act as ceRNAs sponging miR-34a-5p/miR-766-3p/miR-147a/miR-27a-3p, derepressing FGF9 expression; decreased FGF9 promotes EMT via α-SMA and vimentin upregulation	Amplifies fibrotic signaling by inducing tubular EMT, propagating tubulointerstitial fibrosis	Yang et al. (2025)
DKD	Mouse	TGF-β1 mRNA	HG-induced GECs	Podocytes	GEC-derived exosomes transfer TGF-β1 mRNA to podocytes, activating Wnt/β-catenin signaling and inducing podocyte EMT	Transduces endothelial injury signals to podocytes, mediating glomerular fibrosis and dysfunction	Wu et al. (2017)
DKD	Human	FBLN1	HG-induced PTECs	PTECs	Exosomal FBLN1 targets PTECs in an autocrine manner, inducing EMT	Propagates fibrotic signals through autocrine amplification, exacerbating tubular injury	Tsai et al. (2021)
DKD	Mouse	TGF-β1 mRNA	HG-induced GECs	MCs	Transfer of TGF-β1 mRNA activates TGF-β1/Smad3 signaling, promoting MCs activation, proliferation, and ECM production	Transmits pro-fibrotic signals from endothelium to mesangium, driving glomerular ECM accumulation and fibrosis	Wu et al. (2016)
DR	Human	Not specified (proteins, miRNAs)	HG-induced Müller cells	RECs	HG-induced Müller cell exosomes increase intracellular Ca^2+^ in RECs, reducing ZO-1 and TER and disrupting endothelial barrier function	Propagates endothelial barrier dysfunction, contributing to vascular leakage	Gad et al. (2024)
PDR	Mouse	miR-30b	Plasma from PDR mice	RECs	Exosomal miR-30b targets SIRT1 in RECs, promoting angiogenesis	Amplifies pro-angiogenic signaling, driving pathological neovascularization in PDR	Wang et al. (2022b)
DR	Mouse	miR-92a-3p	IL-17A-treated Müller cells	RGCs	IL-17A-stimulated Müller cell exosomes deliver miR-92a-3p to RGCs, targeting Notch-1 and inducing neuronal apoptosis	Propagates neurovascular injury by transmitting apoptotic signals to retinal neurons	Qiu et al. (2025)
PDR	Human	miR-202-5p	HG-induced RPEs (ARPE-19)	HUVECs	Exosomal miR-202-5p targets TGFBR2 in HUVECs, inhibiting TGF/Smad pathway and EMT	Attenuates pathological angiogenesis, counteracting pro-angiogenic signals	Gu et al. (2020)
PDR	Human; mouse	lncRNA-MIAT	Vitreous from PDR patients; HG-induced human RECs	RECs	lncRNA MIAT is enriched under human pathological conditions; The decrease in lncRNA MIAT levels promotes angiogenesis through the lncRNA MIAT/miR-133a-3p/MMP-X1 axis using an oxygen induced retinopathy mouse model	Amplifies pathological neovascularization via pro-angiogenic ceRNA network	Li et al. (2024b)
PDR	Human	miR-9-3p	Müller glia	RECs	Exosomal miR-9-3p targets S1P1 in RECs, activating VEGFR2 phosphorylation and internalization	Promotes pro-angiogenic signaling, driving endothelial activation	Liu et al. (2022a)
DR	Human	miR-21, miR-155, CCL2, MMP2, MMP9	M1-activated microglia	RECs and retinal pericytes	Exosomes deliver pro-inflammatory and pro-angiogenic factors to retinal vascular cells, activating inflammatory and angiogenic pathways	Propagates inflammatory and angiogenic signals, amplifying vascular injury	Beltramo et al. (2023)
DR	Mouse	circEhmt1	Hypoxia-pretreated pericytes	RECs	Exosomes transfer circEhmt1 to RECs, upregulating NFIA and inhibiting NLRP3 inflammasome formation	Maintains vascular homeostasis by transmitting protective signals, attenuating endothelial injury	Ye et al. (2021)
PDR	Human	POSTN	Plasma from PDR patients	—	Exosomal POSTN stabilizes HIF-1α in recipient cells, upregulating angiogenic genes	Amplifies pro-angiogenic signaling, promoting retinal neovascularization	Shen et al. (2025)
PDR	Human	lncRNA LOC100132249	Vitreous from PDR patients	RECs	Exosomal LOC100132249 acts as a ceRNA sponging miR-199a-5p in RECs, derepressing SNAI1 via Wnt/β-catenin pathway and inducing EMT	Amplifies endothelial dysfunction and pro-angiogenic signaling, promoting vascular injury	Hu et al. (2023)
DR	Human	miR-26b-5p	HG-induced HUVEC; serum from DR patients	RECs	Exosomal miR-26b-5p acts on adjacent ECs, regulating endothelial dysfunction	Propagates endothelial injury signals, amplifying vascular dysfunction	Zhang et al. (2022)
Ischemic retinopathy	Retinal astrocytes from human; retinal ganglion cells from mouse	miR-329-5p	Retinal astrocytes	Retinal ganglion cells	miR-329-5p targets MAPK8 (JNK1) mRNA, inhibiting JNK signaling pathway and downstream apoptotic molecules	Suppress Retinal ganglion cells apoptosis, attenuate retinal ischemic injury, provide neuroprotection	Ye et al. (2024)

DKD, diabetic kidney disease; HG, high glucose; MCs, Mesangial Cells; GECs, Glomerular Endothelial Cells; EMT, Epithelial-to-Mesenchymal Transition; TECs, Renal Tubular Epithelial Cells; ceRNA, Competing Endogenous RNA; FGF9, Fibroblast Growth Factor 9; PTECs, Proximal Tubular Epithelial Cells; ECM, extracellular matrix; DR, diabetic retinopathy; RECs, Retinal Endothelial Cells; ZO-1, Zonula Occludens-1; PDR, proliferative diabetic retinopathy; RGCs, Retinal Ganglion Cells; RPEs, Retinal Pigment Epithelial Cells; HUVEC, human umbilical vein endothelial cells; lncRNA, long non-coding RNA; MMP-X1, Matrix Metalloproteinase X1; S1P1, Sphingosine-1-Phosphate Receptor 1; CCL2, C-C Motif Chemokine Ligand 2; MMP2, Matrix Metalloproteinase 2; MMP9, Matrix Metalloproteinase 9; SNAI1, Snail Family Transcriptional Repressor 1; ECs, Epithelial Cells; JNK1, c-Jun N-terminal Kinase 1

Notably, exosomal cargos commonly shared across the pathogenesis of DKD, DR, and DPN remain relatively sparsely identified. A comparative summary of currently recognized common cargos is presented in Table 3, while disease-specific cargo alterations are detailed in Tables 1, 2. Systematic delineation of such shared molecular signatures, encompassing not only common cargo molecules but also conserved cargo-sorting machineries and regulatory pathways, is essential for elucidating the unified mechanisms underlying exosome-mediated crosstalk in diabetic microvascular complications. Given that these complications frequently coexist as comorbidities within the same patient, future investigations should prioritize the identification of convergent exosomal mediators and assembly routes that operate across multiple microvascular beds. Unraveling such shared determinants may ultimately inform the development of multi-target therapeutic strategies capable of simultaneously intervening in multiple diabetic microvascular complications.

**TABLE 3:** Exosomal cargos commonly shared across different diabetic microvascular complications.

Exosome cargo	DKD	DR	DPN
miR-21	M1 macrophage-derived exosomal miR-21a-5p induces podocyte apoptosis (Liang et al., 2022); miR-21-5p targets A20 (TNFAIP3), activates the NLRP3 inflammasome, and promotes podocyte pyroptosis (Ding et al., 2021)	M1 microglia-derived exosomal miR-21 synergizes with CCL2, MMP2/9, disrupting the BRB and inducing pathological angiogenesis (Beltramo et al., 2023)	Exosomal miR-21 from SCs is significantly downregulated under HG, leading to axonal degeneration (Liu et al., 2022b)
miR-221	HG-injured podocyte-derived exosomal miR-221 targets DKK2, relieves inhibition of the Wnt/β-catenin pathway, and induces dedifferentiation of TECs (Su et al., 2020)	Müller cell-derived exosomal lncRNA OGRU acts as a ceRNA sponge for miR-221-3p, upregulates AR and GLUT1, promotes M1 microglial polarization, and exacerbates retinal neuroinflammation and NVU dysfunction (Fu et al., 2024)	Serum exosomal miR-221 targets SOCS3, activates the NF-κB pathway, promotes the release of inflammatory cytokines, and enhances pain hypersensitivity (Wu et al., 2021)
miR-93-5p	M2 macrophage-derived exosomal miR-93-5p targets TLR4, inhibits podocyte apoptosis signaling, exerts renoprotective effects, and attenuates metabolic stress-induced podocyte injury (Wang et al., 2022a)	—	In the diabetic environment, fibroblast-derived exosomal miR-93-5p targets macrophage ATG16L1, inhibits autophagy and activates inflammatory pathways (Xu et al., 2025c). It may indirectly contribute to the modulation of the neuroinflammatory microenvironment
miR-574-5p	Urinary exosomal miR-574-5p levels are correlated with rapid decline in renal function, serving as an early prognostic marker (Barreiro et al., 2025)	Müller cell-derived exosomal lncRNA OGRU acts as a ceRNA sponge for miR-574-5p, upregulates GLUT1 expression, and promotes M1 microglial polarization (Fu et al., 2024)	—
miR-7	Aberrant serum exosomal miR-7 levels are significantly associated with T2DM and its microvascular complications including DKD, serving as a universal screening biomarker (Wan et al., 2017)	—	Serum exosomal miR-7 is associated with early nerve injury in DPN in animal models (Tajabadi et al., 2025)
TGF-β1/TGF-β/Smad Signaling Components	GEC-derived exosomes carry TGF-β1 mRNA, activating the Wnt/β-catenin pathway in podocytes and the TGF-β1/Smad3 pathway in MCs, respectively, inducing podocyte EMT and MC proliferation (Wu et al., 2016; Wu et al., 2017)	Activated RPE-derived exosomes deliver TGF-β/Smad signaling components, induce myofibroblast differentiation, and drive fibrovascular membrane formation and contraction, leading to late-stage complications of PDR (Sreenivas Bk et al., 2025)	—

AR, aldose reductase; BRB, Blood-Retinal Barrier; CCL2, C-C Motif Chemokine Ligand 2; ceRNA, Competing Endogenous RNA; DKD, diabetic kidney disease; DKK2, Dickkopf-related protein 2; DPN, diabetic peripheral neuropathy; DR, diabetic retinopathy; EMT, Epithelial-to-Mesenchymal Transition; GEC, glomerular endothelial cell; GLUT1, Glucose Transporter 1; HG, high glucose; ncRNA, long non-coding RNA; MC, mesangial cell; miR, microRNA; MMP, matrix metalloproteinase; NVU, neurovascular unit; PDR, proliferative diabetic retinopathy; RPE, retinal pigment epithelial cell; SC, schwann cell; SOCS3, Suppressor of Cytokine Signaling 3; T2DM, Type 2 Diabetes Mellitus; TEC, renal tubular epithelial cell; TGF-β, transforming growth factor beta; TLR4, Toll-like receptor 4; TNFAIP3, Tumor Necrosis Factor Alpha-Induced Protein 3

## 4 Diabetic kidney disease

**FIGURE 2:**
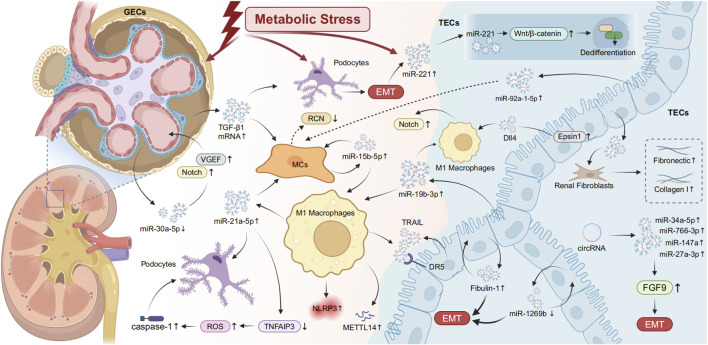
This schematic illustrates how the exosome-mediated pathological network drives glomerular and tubulointerstitial injury in DKD under metabolic stress. Metabolic stress induces target cells, including GECs, podocytes, and MCs, to release exosomes carrying specific miRNA cargos. These exosomes trigger exacerbated inflammation, podocyte EMT, MCs extracellular matrix deposition, and glomerulosclerosis by activating specific signaling pathways. Meanwhile, exosomes secreted by TECs target recipient cells, such as macrophages and renal fibroblasts, and further exacerbate inflammatory infiltration, EMT, and fibrosis via specific proteins and pathways. Collectively, exosomes multi-dimensionally link metabolic dysregulation to the pathological progression of DKD, establishing a core mechanistic framework. GECs, Glomerular Endothelial Cells; VEGF, Vascular Endothelial Growth Factor; MCs, Mesangial Cells; RCN3, Reticulocalbin 3; EMT, Epithelial-to-Mesenchymal Transition; TECs, Renal Tubular Epithelial Cells; Dll4, Delta-like Ligand 4; FGF9, Fibroblast Growth Factor 9.

DKD is one of the most common microvascular complications of diabetes and is a major cause of end-stage renal disease. Metabolic stimuli lead to inflammatory responses and fibrosis, ultimately resulting in a decline in glomerular filtration rate and proteinuria (Alicic et al., 2017; Tuttle et al., 2014). Exosomes play a crucial role in transmitting metabolic damage signals, driving glomerulosclerosis and tubular-interstitial fibrosis (Figure 2).

### 4.1 Metabolic dysregulation and exosome-mediated signaling in DKD

The hyperglycemic environment alters the exosomal secretion and cargo composition in renal cells, transforming metabolic abnormalities into transmissible molecular signals. Exosomes released by TECs under HG conditions show significant upregulation of pro-inflammatory miRNAs, such as miR-19b-3p and miR-92a-1-5p (Lv et al., 2020; Tsai et al., 2023). Furthermore, studies have shown that HG causes phosphorylation and inactivation of the transcription factor FOXO1 in TECs, leading to downregulation of the key GTPase Rab27B, which results in reduced normal exosomal secretion (Zeng et al., 2021). Additionally, TECs stimulated by lipotoxicity or albumin overload release exosomes containing different cargo, such as LRG1 or TRAIL, which activate macrophages or induce TECs apoptosis (Jiang et al., 2022). In MCs, exosomes secreted under HG conditions promote macrophage inflammatory responses (Fujimoto et al., 2025). In GECs, HG-treated GECs release more exosomes, and their content undergoes significant changes, such as an enrichment of mRNA for TGF-β1 (Wu et al., 2016; Wu et al., 2017). HG treatment downregulates miR-30a-5p expression in GECs-derived exosomes, derepressing its inhibitory effect on Notch1 and subsequently activating the Notch1/VEGF signaling pathway, eventually promoting endothelial dysfunction, and aberrant angiogenesis (Ning et al., 2024). At the same time, HG induces the expression of Epsin1 in TECs, specifically promoting the sorting of Dll4 protein into exosomes. These Dll4-containing exosomes, upon uptake by macrophages, effectively activate the Notch1 signaling pathway (Liu JL. et al., 2023). Podocytes also serve as an important source of exosome-mediated signaling in the context of metabolic dysregulation. Under HG conditions, damaged podocytes increase exosomal secretion, with a significant elevation in miR-221 levels. miR-221 in podocyte-derived exosomes directly inhibits the expression of DKK2 and relieves the negative regulation of Wnt/β-catenin signaling, leading to β-catenin accumulation in the nucleus and inducing dedifferentiation of TECs (Su et al., 2020). Changes in the cargo composition of exosomes promote the propagation of pathological signals and the aberrant activation of specific pathways. Collectively, these findings suggest that metabolic dysregulation in DKD dynamically drives disease progression by initiating and remodeling exosome-mediated cell-cell communication.

### 4.2 Glomerular injury and sclerosis

Exosome-mediated cell-cell communication networks play a central role in glomerular injury and sclerosis. Exosomes have recently been recognized as key biomarkers for the early diagnosis of DKD, directly correlating with early glomerular damage. Clinical studies have shown that the level of podocyte transcription factor WT1 protein in urinary-derived exosomes is significantly associated with early declines in estimated Glomerular Filtration Rate (eGFR) and proteinuria in diabetic patients (Abe et al., 2018). Animal experiments further revealed that the elevation of exosomal WT1 precedes the appearance of microalbuminuria (Kalani et al., 2026).

This early abnormal signaling is not isolated but is transmitted and progressively amplified through the exosome network among glomerular cells. First, exosomes derived from GECs serve as crucial messengers initiating podocytes and MCs damage. Exosomes released by GECs under HG conditions carry TGF-β1 mRNA, which, upon internalization by podocytes, activates the Wnt/β-catenin pathway and induces podocyte EMT, directly compromising the filtration barrier (Wu et al., 2017). Simultaneously, these exosomes can be taken up by MCs, where they promote it proliferation and extracellular matrix deposition via the TGF-β1/Smad3 pathway, driving glomerulosclerosis (Wu et al., 2016). As mentioned earlier, damaged podocytes themselves are also active signal senders, promoting the longitudinal transmission of glomerular injury signals to the renal tubules (Su et al., 2020).

Additionally, exosomes derived from immune cells locally amplify inflammation and cell death within the glomerulus. Exosomes from M1 macrophages not only induce podocyte apoptosis but also establish positive feedback communication loops that amplify the damage. Several studies have demonstrated that exosomes secreted by MCs under HG conditions contain miR-15b-5p, which induces apoptosis in both themselves and surrounding cell (Tsai et al., 2020). These exosomes can also be internalized by macrophages, promoting their polarization toward the pro-inflammatory M1 phenotype (Fujimoto et al., 2025). In turn, exosomes released by activated M1 macrophages, which are rich in miR-21a-5p, METTL14, or NLRP3 inflammasome components, can act on MCs and podocyte exacerbating their apoptosis, inflammation, and dysfunction, forming a positive feedback communication loop within the glomerulu (Li Y. et al., 2024; Liang et al., 2022; Liu Y. et al., 2023). Moreover, exosomes activated by HG from macrophages are enriched with miR-21-5p, which targets and inhibits the anti-inflammatory regulator A20 (TNFAIP3), enhancing ROS production and promoting caspase-1 activation, leading to podocyte pyroptosis and locally amplifying the inflammatory response (Ding et al., 2021). These processes transmit and exacerbate the metabolic dysregulation-induced damage to renal microvasculature.

### 4.3 Inflammation and fibrosis in the tubules and interstitium

As DKD progresses, exosome-mediated communication becomes a critical pathological mechanism driving inflammation and fibrosis in the tubules and interstitium. Pathological stimuli first alter the quantity and quality of exosomes secreted by TECs, forming a self-amplifying inflammatory vicious cycle. Damaged TECs release exosomes enriched with factors such as miR-19b-3p, which promote macrophage polarization towards the pro-inflammatory M1 phenotype (Lv et al., 2020; Shen et al., 2021). Activated macrophages subsequently secrete exosomes containing damage signals such as TRAIL, which induce further apoptosis of TECs via the death receptor DR5 pathway, creating a continuously escalating inflammation-apoptosis feedback loop (Jiang et al., 2022). Moreover, exosomes derived from TECs directly stimulate renal fibroblasts, promoting their proliferation and the production of extracellular matrix components such as fibronectin and collagen I (Wen et al., 2020). Exosome-mediated communication also plays a pivotal role in the transmission of fibrosis signals. For instance, exosomes released by TECs under HG conditions can deliver miR-92a-1-5p to MCs in the glomerulus. This miRNA induces endoplasmic reticulum stress by downregulating RCN3, directly driving the transdifferentiation of MCs into myofibroblasts and promoting glomerulosclerosis (Tsai et al., 2023). Simultaneously, exosomes can act on the ECs themselves in an autocrine or paracrine manner. For example, by delivering Fibulin-1 protein or mediating the downregulation of miR-1269b, exosomes induce epithelial-to-mesenchymal transition (EMT), a key event in renal interstitial fibrosis (Tsai et al., 2021). Furthermore, studies have shown that exosomal non-coding RNAs can regulate ferroptosis-related genes through competitive endogenous RNA mechanisms, contributing to the progression of DKD (Liu et al., 2024). Additionally, it has been found that in the DKD environment, exosomes carrying specific circRNAs (e.g., hsa_circ_0006382) can competitively bind miR-34a-5p and other molecules, forming a competing endogenous RNA (ceRNA) network. This network downregulates the expression of target genes such as FGF9, accelerating fibrosis in TECs (Yang et al., 2025). In terms of immune regulation, exosomes derived from CD4^+^ T cells can disrupt mitochondrial homeostasis in TECs, inducing apoptosis via the mitochondrial pathway (Han et al., 2026).

## 5 Diabetic retinopathy

**FIGURE 3:**
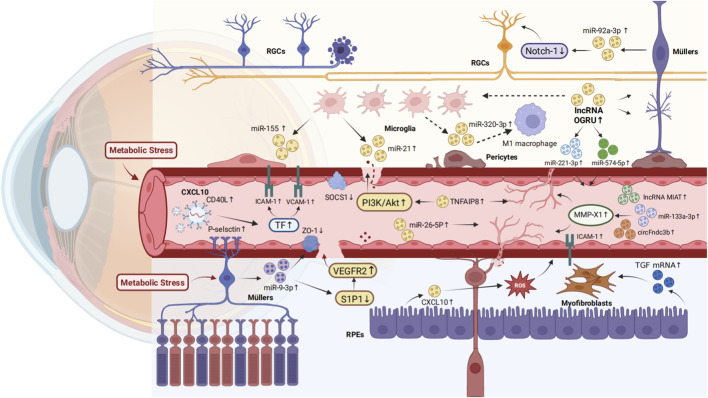
This schematic illustrates how the exosome-mediated pathological network drives neurovascular unit damage in DR under metabolic stress. Metabolic stress induces retinal Müller cells, microglia, pericytes, and other cells to release exosomes carrying specific miRNAs, lncRNAs, and circRNAs. These exosomes disrupt the integrity of the BRB, exacerbate inflammatory responses, and promote pathological neovascularization by regulating key pathways such as Notch-1, PI3K/Akt, and VEGFR2. Meanwhile, exosomal signaling mediates abnormal communication between retinal neurons and glial cells, inducing oxidative stress and fibrosis. Collectively, exosomes multi-dimensionally link metabolic dysregulation to the pathological progression of DR, establishing a core mechanistic framework. BRB, Blood-Retinal Barrier; RGCs, Retinal Ganglion Cells; RPEs, Retinal Pigment Epithelial Cells; ZO-1, Zonula Occludens-1; SOCS, Suppressor of Cytokine Signaling; CXCL10, C-X-C Motif Chemokine Ligand 10; ICAM-1, Intercellular Adhesion Molecule 1; VCAM-1, Vascular Cell Adhesion Molecule 1; MMP-X1, Matrix Metalloproteinase X1; lncRNA, long non-coding RNA.

The pathological mechanisms of DR are driven by a dynamic process involving the dysregulation of a multicellular network within the Neurovascular Unit (NVU). Early stages are marked by the disruption of the BRB, while later stages are characterized by pathological neovascularization (Martins et al., 2020). The core pathology of DR lies in the imbalance of NVU homeostasis. The NVU is composed of retinal microvascular ECs, pericytes, neurons, and glial cells, which together construct and dynamically regulate the BRB through cellular interactions (Gad et al., 2024) (Figure 3)

### 5.1 Metabolic dysregulation and exosome-mediated signaling in DR

Chronic hyperglycemia and its derived pathological products are the primary triggers of retinal oxidative stress and sustained chronic inflammation (Martins et al., 2020). Exosome systems transmit and amplify these pathological signals, driving the disease process.

HG specifically alters the biogenesis and cargo loading of retinal cell-derived exosomes, leading them to carry more pathological components. Studies have shown that exosomes derived from ARPE-19 cells cultured under HG conditions carry increased inflammasome-related cargo and promote the activation of NLRP3 inflammasomes in recipient cells and tissues (Louie et al., 2025). Exosomes from the vitreous of diabetic patients have also been shown to have direct pro-inflammatory effects, significantly upregulating the expression of TNF-α and IL-1β in macrophages, suggesting their role in maintaining a pro-inflammatory microenvironment (Shan et al., 2024). Mechanistic studies indicate that pathogenic exosome generation and release alter the expression of various genes, proteins, and pathways, forming an oxidative damage and inflammation signal transmission network that builds a self-reinforcing feedback loop. HG induces RPEs to release exosomes carrying CXCL10. These exosomes activate the TLR4/MyD88/NF-κB pathway in recipient cells, promoting ROS generation and adhesion molecule expression. Consequently, this exacerbates endothelial oxidative damage and enhances leukocyte infiltration (Zhang et al., 2019). Exosomes released from activated microglia contain significantly upregulated levels of pro-inflammatory miRNAs, such as miR-21 and miR-155 (Beltramo et al., 2023), which target and suppress anti-inflammatory gene expression. Additionally, miR-26b-5p from serum-derived exosomes is significantly upregulated in DR patients and can exacerbate endothelial dysfunction by inhibiting target gene expression. Its levels are positively correlated with DR severity (Zhang et al., 2022). TNFAIP8, a key pro-inflammatory protein carried by exosomes, is also highly expressed in the plasma and vitreous-derived exosomes of DR patients. It can directly promote retinal vascular EC proliferation, migration, and tube formation, with the underlying mechanism being related to the activation of the PI3K/Akt pathway (Xiao et al., 2021).

In addition, various proteins components carried by exosomes also play a crucial role in cell-cell communication of DR. For example, IgG in plasma-derived exosomes can activate the classical complement pathway on the surface of recipient cells, promoting the formation of the Membrane Attack Complex (MAC). In contrast, when exosomes lack IgG, retinal vascular damage in diabetic mice is significantly reduced (Huang et al., 2018). Exosomes derived from retinal astrocytes contain anti-angiogenic proteins such as endostatin, which inhibit angiogenesis (Hajrasouliha et al., 2013). Exosomes from monocytes carry periostin protein, which promotes retinal neovascularization by stabilizing HIF-1α (Shen et al., 2025). This multi-component signaling model enables exosomes to construct and coordinate a complex and finely tuned communication network.

### 5.2 Neurovascular unit dysfunction

Driven by specific communication networks, pathogenic exosomes with functional imbalances disrupt the normal function of the NVU, gradually damaging the BRB. On the vascular side, exosomes released by HG-activated Müller cells, such as miR-9-3p, are efficiently internalized by RECs, leading to downregulation of the tight junction protein ZO-1 and cytoskeletal disruption, impairing the physical structure of the BRB (Liu Y. et al., 2022). Further studies have shown that miR-9-3p in Müller cell-derived exosomes directly targets the S1P1, inhibiting vascular stability signals. miR-9-3p can also activate VEGFR2 phosphorylation, promoting ECs proliferation and vascular leakage (Liu Y. et al., 2022). Meanwhile, pericytes, another key player in vascular stability, are also influenced by the exosome network. Research indicates that MSC-Exos under diabetic conditions downregulate miR-126 in pericytes, which interferes with survival signaling pathways, inducing apoptosis and leading to characteristic pericyte loss (Mazzeo et al., 2015). The disruption of endothelial tight junctions and pericyte apoptosis, mediated synergistically by exosomes, progressively damages the structure and function of the BRB, contributing to retinal edema and hard exudates in clinical pathology (Liu Y. et al., 2022; Mazzeo et al., 2015). On the neural side, exosomes from activated M1 microglia are enriched with pro-inflammatory components that directly induce oxidative damage and functional impairment in photoreceptor cells (Tong et al., 2025). Additionally, exosomes released by activated glial cells participate directly in neuronal damage. Under inflammatory stimuli, exosomes secreted by Müller cells show upregulation of miR-92a-3p, which, upon being internalized by RGCs, targets and suppresses Notch-1 receptor expression, interfering with key pathways for neuronal survival and promoting RGC apoptosis (Qiu et al., 2025). Conversely, a study found that miR-329-5p enriched in exosomes from retinal astrocytes inhibits RGC apoptosis and alleviates ischemia-reperfusion injury by suppressing JNK1, highlighting the bidirectional regulatory role of exosomes in neuronal survival (Ye et al., 2024).

Importantly, exosomes possess the unique ability to coordinate pathological responses across multiple components of the NVU. In the HG environment, exosomes released by Müller cells carry factors that damage both the endothelial barrier and RGCs (Gad et al., 2024). These exosomes also contain substances such as the lncRNA OGRU. Upon uptake by microglia, OGRU adsorbs molecules like miR-320-3p, driving microglial polarization to the pro-inflammatory M1 phenotype, which exacerbates perivascular inflammation and neuronal toxicity (Fu et al., 2024). OGRU can also adsorb miR-221-3p and miR-574-5p, which regulate AR and GLUT1 expression. It can form a multi-target network that cooperatively promotes NVU dysfunction (Fu et al., 2024). This ability of a single exosome subpopulation to coordinate disruptions across vascular, glial, and neuronal compartments underscores the central role of exosomes in early NVU damage in DR.

### 5.3 Retinal ischemia and vascular remodeling

As early NVU damage accumulates, localized retinal capillary occlusion and non-perfused areas develop, leading to progressive ischemia and hypoxia (Joussen et al., 2001; Aiello et al., 1994). Ischemia itself serves as a potent stressor, further altering the exosome secretion profile of cells. These exosomes actively participate in shaping the ischemic microenvironment, then creating a vicious cycle.

Ischemic and hypoxic conditions modify exosome cargo. For example, retinal pericytes cultured under hypoxic conditions release exosomes with significantly upregulated circRNA circEhmt1 expression. Upon internalization by ECs, these exosomes may upregulate NFIA and inhibite NLRP3 inflammasome formation (Ye et al., 2021). The exosome network also exacerbates the ischemic process through multiple pathways. Microparticles released by activated platelets and ECs, rich in TF and phosphatidylserine, provide a catalytic surface for the coagulation cascade, promoting local microthrombus formation and accelerating capillary occlusion (Su et al., 2018). Platelet-derived exosomes are enriched with P-selectin and CD40L, which bind to receptors on monocytes and ECs, triggering the CD40-NF-κB pathway and inducing TF expression (Stępień et al., 2012). This upregulates adhesion molecules such as ICAM-1 and VCAM-1, promoting microthrombus formation and accelerating capillary occlusion (Su et al., 2018). Meanwhile, inflammation signals continuously transmitted via exosomes can induce abnormal synthesis and cross-linking of vascular basement membrane components, leading to diffuse thickening of the basement membrane (Stępień et al., 2012). Additionally, lncRNA DLX6-AS1, highly expressed in serum-derived exosomes from DR patients, regulates target gene expression to promote vascular smooth muscle cell proliferation and basement membrane synthesis, accelerating vascular remodeling (Ye et al., 2022).

### 5.4 Pathological neovascular proliferation and fibrosis

When retinal ischemia reaches a critical threshold, the disease progresses into the proliferative phase, marked by the formation of pathological neovascularization. Exosome-mediated cell-cell communication plays a key role in this stage by transmitting pro-angiogenic signals that drive the pathological process.

Exosomes released by various cells in ischemic retina contribute significantly to pathological neovascular formation. For instance, exosomes isolated from the plasma of Proliferative Diabetic Retinopathy (PDR) patients show elevated levels of miR-30b. Upon internalization by ECs, it targets and inhibits the deacetylase SIRT1, relieving the suppression of EC proliferation (Wang P. et al., 2022). Studies of vitreous exosomes from PDR patients revealed significant enrichment of the lncRNA LOC100132249, which acts as a competitive endogenous RNA for miR-199a-5p, leading to upregulation of the pro-angiogenic gene SNAI1 (Hu et al., 2023). Additionally, lncRNA MIAT carried by exosomes enhances EC migration and tube formation by adsorbing miR-133a-3p and upregulating MMP-X1 expression, while circFndc3b from serum-derived exosomes promotes neovascularization by regulating angiogenesis signaling pathways (Li X. et al., 2024; Li et al., 2021). Exosomes also mediate the influence of systemic factors on local neovascularization. HG enhances glycolytic metabolism in monocytes, causing histone lactylation modifications that upregulate the transcription of the pro-angiogenic extracellular matrix protein osteopontin (POSTN). Monocytes then release POSTN-enriched exosomes into circulation, and upon reaching the ischemic retina, these exosomes stabilize HIF-1α, maintaining a pro-angiogenic program (Shen et al., 2025). Studies have confirmed that POSTN levels in exosomes from patient plasma are significantly elevated, and metformin can reduce POSTN secretion by inhibiting monocyte glycolysis (Shen et al., 2025).

Furthermore, exosomes participate in the fibrotic remodeling of the fibrovascular membrane. Exosomes released by activated RPEs deliver TGF-β/Smad signaling components, activating myofibroblast differentiation and promoting excessive extracellular matrix deposition, which leads to fibrovascular membrane formation and contraction (Sreenivas Bk et al., 2025). Additionally, exosomes from platelet-rich plasma activate YAP via the PI3K/Akt pathway, enhancing Müller cell fibrosis activity and accelerating proliferative membrane formation (Zhang et al., 2020).

## 6 Diabetic peripheral neuropathy

**FIGURE 4:**
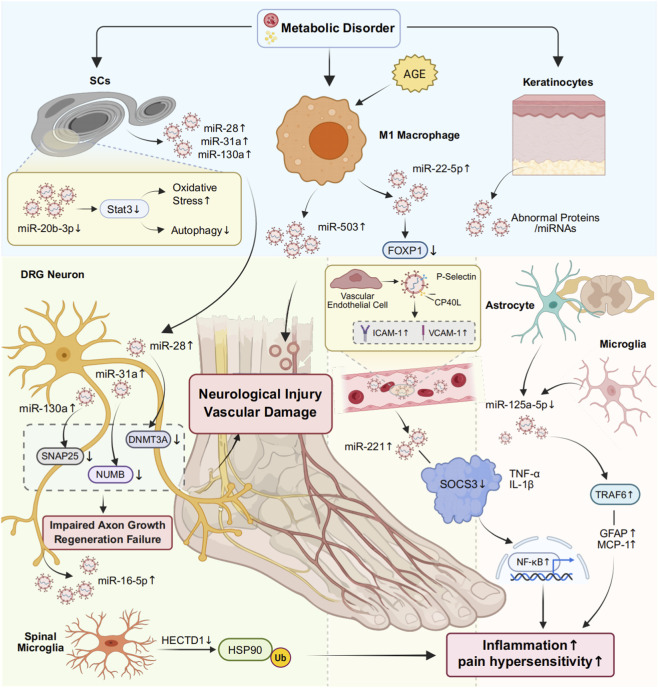
This schematic illustrates how the exosome-mediated pathological network drives neurological and vascular damage in DPN. Under metabolic stress, SCs, M1 macrophages, and keratinocytes release exosomes carrying specific miRNA cargos, which propagate injury signals to DRG neurons, vascular ECs, and spinal microglia. In DRG neurons, dysregulated miRNAs target specific genes, impairing axon growth and regeneration. In the vascular system, exosomes exacerbate endothelial dysfunction and vascular damage by upregulating adhesion molecules and other pathways. Meanwhile, exosomal miRNAs amplify inflammatory responses and pain hypersensitivity by transmitting pro-inflammatory signals. Collectively, this diagram provides a mechanistic framework for understanding how exosomal signaling links metabolic dysregulation to DPN progression. SCs, Schwann Cells; AGE, Advanced Glycation End Products; FOXP1, Forkhead Box Protein P1; ICAM-1, Intercellular Adhesion Molecule 1; VCAM-1, Vascular Cell Adhesion Molecule 1; SNAP25, Synaptosomal-Associated Protein 25; NUMB, NUMB Endocytic Adaptor Protein; DNMT3A, DNA methyltransferase-3α; SOCS, Suppressors of Cytokine Signaling; GFAP, Glial Fibrillary Acidic Protein; MCP-1, Monocyte Chemoattractant Protein-1; TRAF6, TNF Receptor-Associated Factor 6.

DPN is a common complication of diabetes, characterized by damage to the peripheral nerves, leading to sensory, motor, or autonomic dysfunction (Zhang L. et al., 2025). The pathological process of DPN involves the interplay of multiple factors, including metabolic dysregulation, oxidative stress, inflammation, and vascular dysfunction (Hicks and Selvin, 2019). Increasing evidence suggests that, within the pathological environment of DPN, metabolic dysregulation also remodels exosome biogenesis and cargo loading (Jia et al., 2018). These abnormally programmed exosomes form a specific communication network that connects SCs, neurons, ECs, and immune cells, amplifying the damage signals in a coordinated manner (Figure 4).

### 6.1 Metabolic dysregulation and exosome-mediated signaling in DPN

In the progression of DPN, metabolic dysregulation also drives the exosome-mediated pathological signaling network. First, HG environments directly induce functional impairment and phenotypic transformation in key cells, leading to the release of exosomes carrying pathological signals. Exosomes derived from healthy SCs deliver protective miR-21, which promotes nerve growth by activating the AKT pathway, and significantly improves nerve function and myelin regeneration in DPN animal models (Liu YP. et al., 2022; Wang et al., 2020). In contrast, SCs exposed to HG show inhibited proliferation, increased apoptosis, and secrete exosomes enriched with miRNAs such as miR-28, miR-31a, and miR-130a (Jia et al., 2018). These exosomes significantly inhibit axonal growth *in vitro*. Notably, miR-20b-3p targets Stat3 in SCs, and its loss or downregulation leads to autophagy blockage and increased oxidative stress, initiating SCs dysfunction (Li J. et al., 2023). Similarly, ECs and immune cells in a HG environment also exhibit significant functional abnormalities. HG induces macrophage polarization to the pro-inflammatory M1 phenotype, and the exosomes they release show upregulated expression of miR-503, which targets IGF1R to inhibit EC function (Wang et al., 2023b).

Ultimately, these reprogrammed pathological exosomes exhibit abnormal targeting and cell-cell communication abilities, leading to their aberrant uptake by distant cells. Exosomes from keratinocytes show significant differences in protein and miRNA content and reverse the abnormal molecular cargo to DRG neurons, impacting axonal integrity, synaptic function, and local inflammatory responses (Coy-Dibley et al., 2024; Coy-Dibley et al., 2025). Exosomes derived from SCs can be internalized by DRG neurons, affecting their nerve growth (Liu YP. et al., 2022). When DRG neuron-derived exosomes are taken up by spinal microglia, the enriched miR-16-5p targets and inhibits HECTD1, alters HSP90 ubiquitination, and drives microglial pro-inflammatory activation, consequently amplifying neuroinflammation and exacerbating pain sensitization (Xing et al., 2024). Additionally, studies have also found that fibroblast-derived exosomes from diabetic environments can deliver miR-93-5p to target macrophage autophagy and inflammation pathways, indirectly influencing the neuroinflammatory microenvironment (Xu Z. et al., 2025). In conclusion, this process extends from local microenvironmental deterioration to interactions between distal neurons and immune cells, ultimately driving the progression of neuropathy.

### 6.2 Roles of exosome-mediated cell-cell communication in different stages of DPN

In the progression of DPN, exosome-mediated cell-cell communication plays a dynamic role in mediating and amplifying neuronal injury signals at various pathological stages. In the early stages, HG conditions reprogram exosome cargo, directly initiating axonal damage. SCs, as the primary glial cells of peripheral nerves, secrete exosomes that undergo significant cargo alterations, as previously discussed.

When exosomes derived from HG-stimulated SCs are internalized by axons of DRG neurons, they inhibit the expression of DNA methyltransferase-3α, NUMB, and SNAP25, directly impairing axonal growth, regeneration, and causing early degeneration (Jia et al., 2018). This process constitutes the core molecular event of early axonal degeneration in DPN. Animal models further validated the *in vivo* effects of these pathological exosomes. For instance, when exosomes from HG-stimulated SCs were injected into the sciatic nerves of db/db mice, they accelerated the decline in nerve conduction velocity, decreased mechanical and thermal sensation, and were accompanied by reduced epidermal nerve fiber density (Jia et al., 2018). Exosome networks also transmit inflammatory signals, amplifying neuropathic pain. As the disease progresses, inflammation becomes a key driver of DPN, particularly in painful diabetic neuropathy. In DPN patients, miR-221 expression in serum-derived exosomes is upregulated and activates the NF-κB pathway by targeting SOCS3, promoting the release of inflammatory cytokines and further enhancing pain hypersensitivity (Wu et al., 2021). In addition to reverse transport to the DRG, exosomes derived from keratinocytes participate in the generation and maintenance of pain signals (Coy-Dibley et al., 2024; Coy-Dibley et al., 2025). Exosomes from astrocytes, enriched with miR-125a-5p, can regulate neuroinflammation by targeting TRAF6 (Kasimu et al., 2022). Downregulation of miR-125a-5p leads to increased expression of GFAP and MCP-1, further amplifying pain responses (Kasimu et al., 2022).

As the disease advances, exosome-mediated communication between the vascular and neural units leads to a vicious cycle (Zhong et al., 2025). Damaged SCs further release their pathological exosomes, with profound changes in their cargo composition. Notably, miR-28 and miR-31a levels significantly increase (Jia et al., 2018). When these exosomes act on ECs, miR-28 directly targets and inhibits DNMT3a, while miR-31a targets and inhibits the endocytosis adaptor protein NUMB (Jia et al., 2018). The dysregulation of these molecules leads to a decrease in the expression and redistribution of tight junction proteins in ECs, directly compromising the structural integrity of the blood-nerve barrier. These processes also exacerbate local inflammation and neuroischemia (Jia et al., 2018). This ischemic and inflammatory microenvironment, in turn, stimulates ECs and SCs to produce and release more exosomes enriched with damage signals, forming a vicious cycle (de Jong et al., 2012). Exosomes released by monocytes and ECs under HG conditions can also mutually activate this cycle, driving the upregulation of ICAM-1 of EC and enhancing monocyte adhesion, leading to impaired microcirculatory perfusion and increased barrier vulnerability (de Jong et al., 2012; Ji et al., 2025). Additionally, AGE-stimulated macrophages release exosomes enriched with miR-22-5p, which target and inhibit FOXP1, leading to decreased eNOS phosphorylation and a significant increase in monocyte adhesion (Ji et al., 2025). Thus, as DPN progresses, exosomes no longer serve as unidirectional signal carriers but become a multidirectional communication hub integrating neural, vascular, and immune cell signaling. The signal amplification mediated by these exosomes leads to the mutual exacerbation of nerve dysfunction and microcirculatory impairment, driving the disease into an irreversible vicious cycle.

## 7 Clinical implications of exosome-mediated cell-cell communication: diagnosis and potential therapeutic strategies

### 7.1 Exosomes as diagnostic biomarkers

Early diagnosis of diabetic microvascular complications remains a significant clinical challenge. Early intervention is crucial for improving long-term patient outcomes. Exosomes, as emerging carriers of biomarkers, offer promising new avenues for achieving this goal. Exosomes in the circulatory system, particularly those in serum or plasma, have demonstrated diagnostic potential for various complications. In DKD, serum-derived exosomes exhibit a distinct miRNA profile, with upregulation of miR-1246, let-7c-5p, and other miRNAs correlating with proteinuria severity (Kim et al., 2019). Additionally, elevated AEBP1 mRNA levels in plasma extracellular vesicles effectively distinguish DKD patients from those with simple diabetes (Tao et al., 2021). In DR, changes in exosomal proteins such as LGALS3, MYH10, CPB2 (Wang et al., 2024), and RBP3 have been identified as potential biomarkers for DR diagnosis and severity grading (Gr et al., 2025). For DPN, there is currently no clear evidence from human studies identifying reliable exosomal biomarkers for diagnosis. However, previous studies in animal models have found dysregulation of miR-7 and miR-221, which may be associated with early detection of DPN, warranting further investigation (Tajabadi et al., 2025). Compared to blood, urinary-derived exosomes have the advantage of DKD diagnosis. For example, elevated levels of PAK6 proteins in urinary-derived exosomes correlate negatively with kidney function and provide important diagnostic value (Li T. et al., 2023). Transcription factor Elf3, released from damaged podocytes, can be directly detected in urinary-derived exosomes and serves as a marker for the rate of kidney function decline (Sakurai et al., 2019). This non-invasive diagnostic approach has been extended to other complications, with specific enrichment of JUP in urinary-derived exosomes from DR patients (Mighty et al., 2022). Additionally, some studies have suggested that exosomes derived from tears may have diagnostic value in DR (Mighty et al., 2022). These findings lay the foundation for a multi-level exosome diagnostic system, where exosomes accurately encapsulate and reflect the pathological status of their source cells, enabling the assessment of damage to different microvascular target organs through analysis of exosome cargo in bodily fluids (Table 4).

**TABLE 4:** Exosomes in the diagnosis of diabetic microvascular complications.

Disease background	Source	Exosomal cargo	Correlation with disease progression	References
DKD	Serum	microRNAs (miR-1246, let-7c-5p, miR-642a-3p, miR-1255b-5p, let-7i-3p, miR-5010-5p, miR-150-3p, miR-4449)	Significantly correlated with the degree of albuminuria	Kim et al. (2019)
DKD	Plasma	AEBP1 mRNA	Positively correlated with Cr, 24-h urine protein, serum CYC; negatively correlated with eGFR and LDL.	Tao et al. (2021)
DKD	Blood	WT1 mRNA, ACE mRNA	WT1 positively correlated with urine Alb/Cr ratio; ACE negatively correlated with urine Alb/Cr ratio	Hashemi et al. (2021)
DKD	Plasma	uracil, LPC(O-18:1/0:0), sphingosine 1-phosphate, 4-acetamidobutyric acid	Associated with the occurrence and progression of DKD; potential early diagnostic biomarkers	Pan et al. (2022)
DKD	Serum	miR-1207-5p	Inversely associated with parameters of renal dysfunction; may confer protection against DKD progression	Zhou et al. (2025)
DKD	Urine	WT1 mRNA	Reflects podocyte injury; predicts decline in eGFR; correlates with albuminuria and eGFR.	Abe et al. (2018)
DKD	Urine	miR-145-5p, miR-27a-3p	Positively correlated with albuminuria and serum creatinine; negatively correlated with eGFR.	Han et al., 2024)
DKD	Urine	miR-615-3p	Positively correlated with serum Cystatin C, plasma TGF-β1, creatinine, BUN, PCR, 24-h urine protein; negatively correlated with eGFR and albumin	Wang et al. (2023c)
DKD	Urine	miR-192-5p, miR-146a-5p, miR-486-5p, miR-574-5p	Classify individuals with fastest kidney function decline even in normoalbuminuria group	Barreiro et al. (2025)
DKD	Urine	miR-15b-5p	Levels correlated with low baseline kidney function and rapid decline in kidney function	Tsai et al. (2020)
DKD	Urine	miR-200b	Decreased with fibrosis progression; correlates with severity of renal fibrosis	Yu et al. (2018)
DKD	Urine	miR-362-3p, miR-877-3p, miR-150-5p, miR-15a-5p	Potential biomarkers for incipient DKD; may regulate pathways involved in DKD.	Xie et al. (2017)
DKD	Urine	CKAP4 protein	Positively correlated with glomerulosclerosis, interstitial fibrosis and tubular atrophy	Zhang et al. (2025b)
DKD	Urine	Elf3 protein	Correlated with the rate of decline in eGFR; potential early marker for podocyte injuries	Sakurai et al. (2019)
DKD	Urine	UMOD mRNA	Progressively elevated from T2DM to DKD groups; correlates with eGFR and ACR.	Yamamoto et al. (2018)
DR	Plasma	LGALS3, MYH10, CPB2	Potential biomarkers for DR diagnosis and severity grading	Wang et al. (2024)
DR	Plasma	CELA3A, CELA3B, CTRC	Correlated with DR progression and are promising biomarkers for disease staging and progression monitoring	Li et al. (2025)
DR	Plasma	RBP3	Levels decrease with DR progression, serving as a potential diagnostic biomarker for PDR.	Gr et al. (2025)
DR	Urine	JUP	Potential disease biomarkers for DR.	Mighty et al. (2022)
PDR	Vitreous humor	LDHA, Ficolin-3, APOB, APOM	Potential disease biomarkers and therapeutic targets for PDR.	Wang et al. (2023d)
PDR	Vitreous humor	miR-125 family (downregulated), miR-21-5p (upregulated)	Potential disease biomarkers for PDR.	Kot and Kaczmarek (2022)
DR	Tears	ODF2L, TSR1, TONSL, FUT6 mRNAs	Potential disease biomarkers for DR.	Chen et al. (2025a)
PDR	Serum	miR-431-5p	Potential biomarkers for disease progression in PDR.	Yu et al. (2021)
DME	Serum	miR-377-3p	Inhibits VEGF expression and provides a diagnostic biomarker for DME.	Jiang et al. (2021)
T2DM and its Microvascular Complications	Serum	miR-7	Significantly associated with T2DM and its microvascular complications, showing potential as a promising biomarker	Wan et al. (2017)

DKD, diabetic kidney disease; CYC, cystatin-C; eGFR, estimated Glomerular Filtration Rate; LDL, Low-Density Lipoprotein; Alb, Albumin; Cr, Creatinine; BUN, blood urea nitrogen; PCR, Protein-to-Creatinine Ratio; ACR, Albumin-to-Creatinine Ratio; DR, diabetic retinopathy; PDR, proliferative diabetic retinopathy; DME, Diabetic Macular Edema; T2DM, Type 2 Diabetes Mellitus; VEGF, vascular endothelial growth factor.

It should be noted, however, that the clinical translation of the candidate biomarkers discussed above still faces several important challenges. Most available studies are single-center, cross-sectional investigations with small sample sizes. In many cases, key confounding factors, including age, disease duration, renal function, and concomitant medication use, have not been adequately controlled (Wang et al., 2024; Zheng et al., 2024). In addition, substantial heterogeneity exists in exosome isolation approaches, such as ultracentrifugation, size-exclusion chromatography, and immunoaffinity capture, as well as in the detection platforms used across studies. This methodological variability can directly influence the quantification of downstream miRNAs and weaken comparability between studies (Théry et al., 2018; Welsh et al., 2024). Pre-analytical factors also remain insufficiently standardized, including the timing of urine collection, urine dilution, and the number of freeze-thaw cycles (Welsh et al., 2024). Although some biomarkers have shown encouraging performance in discovery cohorts, independent external validation is often lacking. Moreover, the pooled diagnostic sensitivity and specificity reported to date remain only moderate (Zheng et al., 2024), suggesting that multi-marker panels may be more informative than single molecules (Li T. et al., 2023). Future clinical studies should therefore follow minimal reporting standards. At a minimum, these should include detailed information on participant inclusion and exclusion criteria, disease stage, sample collection and pre-processing, exosome isolation and characterization procedures, detection platforms, as well as normalization strategies (Théry et al., 2018; Welsh et al., 2024; Bossuyt et al., 2015; Lucien et al., 2023; Van Deun et al., 2017; Bustin et al., 2009). Key diagnostic accuracy measures, including predefined thresholds, AUC, sensitivity, specificity, and results from independent validation, should also be clearly reported (Bossuyt et al., 2015). On this basis, multicenter prospective cohort studies and integrative models that combine exosome-based multi-marker panels with conventional clinical parameters will be essential for moving exosome biomarkers closer to routine clinical use.

### 7.2 Therapeutic strategies with stem cell-derived exosomes

Stem cells and their derived exosomes represent an emerging strategy with significant potential in treating diabetic microvascular complications. Their core value lies in their ability to mimic the regenerative function of stem cells by precisely delivering bioactive molecules to damaged target cells, regulating key pathological processes at multiple targets. In various complications, stem cell-derived exosomes exert therapeutic effects through common yet tissue-specific mechanisms. In DKD, ADSCs-derived exosomes deliver miR-215-5p to inhibit podocyte ZEB2 expression, alleviating EMT (Jin et al., 2020). It can also carry deubiquitinase USP25 to stabilize SMAD7 and suppress TGF-β signaling, reducing podocyte apoptosis and inflammation (Wang X. et al., 2025). Bone marrow-derived mesenchymal stem cells (BMSCs)-derived exosomes primarily mitigate renal cell injury and fibrosis by inhibiting NF-κB and apoptosis-related proteins (Liu L. et al., 2023). These actions collectively aim to reduce inflammation, inhibit abnormal cell death, and reverse fibrosis.

In the more complex pathological mechanisms of DR, the application of stem cell-derived exosomes is more extensive. Firstly, in terms of alleviating neuroinflammation, MCSs-derived exosomes deliver miR-22-3p or miR-29a-3p to microglial cells, which respectively inhibit the NLRP3 inflammasome and activate the HMGB1/TLR4 pathway, promoting the polarization of microglial cells toward the M2 phenotype (Tong et al., 2025; Chen Y. et al., 2024). Second, exosomes engineered to deliver USP25 to photoreceptors inhibit their apoptosis (Sun et al., 2025), while BMSCs-derived exosomes carrying miR-483-5p target IGF-1R in RPEs to suppress HG-induced apoptosis (Cao et al., 2024). Finally, regarding pathological angiogenesis, stem cell-derived exosomes exert their effects through various mechanisms. For instance, exosomes from human umbilical mesenchymal stem cells (MSCs) deliver miR-18b, targeting MAP3K1 to exert anti-angiogenic effects (Xu et al., 2021). More advanced strategies include loading anti-angiogenic drugs, such as pigment epithelium-derived factor (PEDF) or bevacizumab, into exosomes to create efficient biological delivery systems (Fan et al., 2023; Reddy et al., 2023). In contrast, research on stem cell-derived exosome therapies for DPN is limited but shows clear mechanisms, such as MSC-Exos reducing neuroinflammation by inhibiting TLR4/NF-κB signaling (Fan et al., 2020), or promoting angiogenesis and improving nerve function by delivering miR-130a-3p to SCs (Chen et al., 2023).

In summary, stem cell-derived exosomes construct a novel, beneficial cell-cell communication network by precisely delivering specific miRNAs, proteins, or drugs to damaged cells in the kidneys, retina, or peripheral nerves (Yao et al., 2026; Hao et al., 2026). By simultaneously targeting anti-inflammatory, anti-apoptotic, anti-fibrotic, and anti-angiogenic processes at multiple pathological points. These exosomes provide a strong theoretical foundation and transformative potential for developing common therapeutic approaches for multiple diabetic microvascular complications.

### 7.3 Therapeutic strategies with classic antidiabetic drugs and natural products

The therapeutic effects of classic antidiabetic drugs and emerging natural products are being revisited. Beyond their traditional mechanisms of lowering blood glucose, blood pressure, and lipids, preliminary evidence suggests that these interventions can also regulate the exosome cargo and modulate exosome release and uptake, influencing the abnormal cell-cell communication involved in the progression of diabetic microvascular complications. This perspective extends the multifunctional protective roles of commonly used drugs to a new molecular interface and offers a breakthrough for understanding the therapeutic targets of natural products.

Metformin is the most studied drug in this context. In the field of microvascular complications, metformin confers renal protection through the modulation of exosomes. One study found that metformin significantly reduced the expression of cathepsin B in the kidney tissue of diabetic mice, and the abundance of cathepsin B in exosomes derived from urine decreased accordingly. Exosomes rich in cathepsin B can directly enhance sodium channel activity in the collecting duct epithelium. Metformin regulates blood pressure and delays kidney disease progression by inhibiting this exosome-mediated communication (Scindia et al., 2023). Although there is no evidence yet regarding the regulation of exosome-mediated communication in DR or DPN models, innovative studies on metformin in other diabetic comorbidities provide insight. For example, metformin-pretreated BMSCs-derived exosomes competitively bind to miR-139-3p, relieving the inhibition of FOXC2 and leading to enhanced EC tube formation and wound healing (Gu et al., 2025). Metformin treatment was also found to correlate with the downregulation of exosome miRNAs such as miR-24 and miR-27, which are related to inflammation (Sardu et al., 2021).

Glucagon-like peptide-1 receptor agonists (GLP-1RAs) have also been shown to exert multifaceted protection through exosome pathways beyond glucose-lowering effects. Clinical studies have demonstrated that liraglutide combined with metformin significantly reduces the concentration of total, endothelial-derived, and platelet-derived exosomes in the circulation of diabetes patients. This suggests that it has unique pharmacological properties that directly improve EC activation and reduce the release of pro-inflammatory extracellular vesicles (Baldassarre et al., 2026). In a DKD podocyte injury model, HG-induced APOC1 in podocytes is delivered via exosomes to macrophages, inducing M1 polarization through the HOXD9/APOC1/NF-κB pathway. Liraglutide treatment partially reverses this process, and knocking out exosomal APOC1 enhances the anti-inflammatory effects of liraglutide (Feng et al., 2024). In DR and endothelial dysfunction models, semaglutide inhibits the accumulation of miR-155 in lipopolysaccharide-induced macrophage exosomes, relieving miR-155s inhibition of EPCs activity, migration, and tube formation (Pan et al., 2023).

A limited number of studies have also focused on sodium-glucose cotransporter-2 inhibitors (SGLT2i) from this perspective. One study found that ADSCs-derived exosomes treated with empagliflozin significantly promoted human umbilical vein endothelial cells (HUVEC) proliferation, migration, and tube formation by activating the PTEN/AKT/VEGF signaling pathway (Wang H. et al., 2025). Another study in a myocardial ischemia-reperfusion injury model demonstrated that empagliflozin pretreatment increased the secretion of BMSCs-derived exosomes, which carried mitochondrial regulatory protein ATAD3A. These exosomes can activate PINK1/Parkin-mediated mitophagy, providing insights for treating microvascular diseases (Jing et al., 2025).

In addition to classic antihyperglycemic agents, natural products and their active components have recently shown significant potential in treating diabetic microvascular complications by modulating exosome-mediated pathological communication. Existing studies have identified three main pathways through which natural products regulate exosome networks. Natural products can directly act on key cells within the affected microenvironment, reshaping their exosomal cargo and blocking pathological signal transmission. For example, Astragaloside IV (ASIV), a primary active component of astragalus, has been shown to promote the secretion of exosomes from endothelial progenitor cells (EPCs). The ASIV-treated EPC-derived exosomes carry miR-21, which enhances autophagy and inhibits apoptosis of ECs under hyperglycemic conditions (Xiong et al., 2022). Additionally, studies have found that fenugreek extract may reduce the activity of arginine transported by serum exosomes, potentially preventing ECs uptake and the subsequent elevation of arginine ester levels (Qiu et al., 2024).

MSC-Exos have inherent repair potential, which can be further amplified by preconditioning with natural products. ASIV, when used to precondition MSCs, enhances the secretion of exosomes enriched with pro-angiogenic miR-146a-5p (Chen et al., 2025b). Loading active drugs into exosomes significantly improves their bioavailability and therapeutic index. For instance, curcumin-loaded macrophage exosomes promote HUVEC proliferation, migration, and angiogenesis *in vitro*, while reducing ROS levels, modulating mitochondrial membrane potential, and alleviating oxidative stress and inflammation (Li D. et al., 2023).

Plant-Derived Exosome-like Nanoparticles (PENPs) have emerged as a novel natural nanoplatform, attracting growing attention. Unlike synthetic carriers, PENPs serve dual functions as both delivery vehicles and innate bioactive agents. Their naturally carried lipids, proteins, miRNAs, and active saponins confer antioxidant, anti-inflammatory, and metabolic regulatory properties (Xiao et al., 2025). Ginseng-Derived Exosomes (GExos) are rich in ginsenosides (Rg1, Re, Rb1) and functional miRNAs (Tan et al., 2024). Mechanistic studies have shown that GExos significantly upregulate key glycolytic enzymes (PFKM, PGK1, and ENO1), leading to enhance ATP production and improving endothelial energy metabolism (Tan et al., 2024). Lemon-derived PENPs also exhibit significant immunomodulatory activity. When incorporated into functionalized hydrogel matrices, lemon-derived PENPs reduce local inflammation, improve tissue perfusion, and modulate macrophage polarization by inhibiting the NF-κB signaling pathway and promoting M2 polarization (Jin et al., 2025). Although many of these studies focus on wound healing in diabetic ulcers, their therapeutic effects on vascular injury provide important insights for the innovation of interventions for diabetic microvascular complications. Despite their promising translational potential, PENPs remain constrained by several major knowledge gaps: the absence of standardized, scalable manufacturing and quality-control workflows; substantial batch-to-batch heterogeneity stemming from variations in plant source and processing conditions; and an incomplete understanding of their *in vivo* pharmacokinetics, biodistribution, metabolism, and clearance profiles. Addressing these gaps will be essential for advancing PENPs toward clinical application (Jo et al., 2025; Zheng et al., 2025) (Table 5)

**TABLE 5:** Targeting exosome-mediated cell communication for the treatment of diabetic microvascular complications.

Disease type	Intervention model	Intervention method	Mechanism of action	References
DKD	LPS-induced podocyte injury	M2 macrophage-derived exosomes	Exosomal miR-93-5p targets TLR4 to inhibit podocyte apoptosis, exerting renoprotective effects	Wang et al. (2022a)
DPN	HG-induced SCs	miR-20b-3p agomir	Exosomal miR-20b-3p targets Stat3 to reduce p-Stat3 levels, restoring autophagic flux in SCs and improving nerve function	Li et al. (2023a)
DPN	HG-induced astrocytes	miR-125a-5p mimic	miR-125a-5p targets TRAF6 to downregulate GFAP and MCP-1, attenuating neuroinflammation and pain hypersensitivity	Kasimu et al. (2022)
DKD	HG-induced mouse podocytes	ADSCs-derived exosomes	Transfer miR-215-5p through ADSCs-derived exosomes to podocytes, suppressing ZEB2 transcription and alleviating HG-induced EMT and podocyte migration	Jin et al. (2020)
DKD	HG-induced mouse podocytes	ADSCs-derived exosomes	Transfer USP25 to podocytes through ADSCs-derived exosomes. USP25 interacts with SMAD7 and induces its deubiquitination, stabilizing SMAD7 and suppressing podocyte apoptosis and inflammation	Wang et al. (2025b)
DKD	HFD-induced rats	BMSCs-derived exosomes	Alleviate renal injury through the inhibition of apoptosis and inflammation, as decreased Cleaved Caspase-3/9 expression, reduced TNF-α transcription, and diminished NF-κB p65 expression	Liu et al. (2023c)
DKD	HG-induced HK-2 cells	BMSCs-derived exosomes	Transfer miR-30e-5p to inhibit caspase-1-mediated pyroptosis by targeting ELAVL1	Lv et al. (2023)
DR	STZ and HFD-induced diabetic rats	MSC-Exos	Alleviates DR by delivering miR-22-3p to inhibit NLRP3 inflammasome activation	Chen et al. (2024b)
DR	STZ-induced diabetic rats	MSC-Exos	Attenuates DR by delivering miR-29a-3p to regulate microglia M1 polarization via targeting HMGB1	Tong et al. (2025)
DR	db/db mice	Engineered photoreceptor-targeting peptide (MH42)-modified, USP25-enriched MSC-Exos	Alleviates DR by targeted delivery of deubiquitinating enzyme USP25 to inhibit photoreceptor apoptosis	Sun et al. (2025)
DR	HG-induced ARPE-19 cells	BMSCs-derived exosomes	Suppresses HG-induced apoptosis by delivering miR-483-5p to target IGF-1R	Cao et al. (2024)
DR	STZ-induced diabetic rats	Human umbilical cord MSC-Exos	Exerts anti-inflammatory and anti-apoptotic effects by transmitting miRNA-18b to target MAP3K1 and inhibit NF-κB p65 phosphorylation	Xu et al. (2021)
Retinopathy	Oxygen-induced retinopathy mice	PEDF-loaded MSC-Exos	Enhances anti-angiogenic, anti-inflammatory, and neuroprotective effects of PEDF by increasing its stability and penetrability	Fan et al. (2023)
DR	STZ-induced DR rats	Bevacizumab-loaded MSC-Exos	Acts as a drug delivery vehicle to prolong the therapeutic effect of bevacizumab and reduce the frequency of intravitreal injections	Reddy et al. (2023)
DPN	db/db mice	MSC-Exos	Alleviates neurovascular dysfunction and improves functional recovery in DPN by targeting the TLR4/NF-κB signaling pathway, inhibiting pro-inflammatory gene expression, and modulating macrophage phenotype	Fan et al. (2020)
DPN	STZ-induced DPN rats	miR-130a-3p-carrying ADSCs-derived Exosomes	Alleviates DPN by delivering miR-130a-3p to downregulate DNMT1, activating the NRF2/HIF-1α/ACTA1 axis, promoting SCs proliferation, inhibiting apoptosis, and enhancing angiogenesis	Chen et al. (2023)
DKD	Salt-loaded hypertensive diabetic db/db mice	Metformin	Lowers blood pressure and delays nephropathy progression by reducing renal Cathepsin B expression. Urinary exosomal Cathepsin B may serve as a non-invasive biomarker reflecting this mechanism	Scindia et al. (2023)
Diabetic Wound	Diabetic mouse wound model	Empagliflozin-pretreated MSC-Exos	Accelerates diabetic wound healing by activating the PTEN/AKT/VEGF signaling pathway, significantly enhancing the proliferation, migration, invasion, and angiogenesis of HUVECs	Wang et al. (2025c)
Diabetic endothelial dysfunction	HG-induced HUVECs	Astragaloside IV-pretreated EPC-derived exosomes	Promotes autophagy and inhibits HG-induced ECs apoptosis by delivering miR-21 to target PTEN, thereby activating the PI3K/AKT/mTOR pathway	Xiong et al. (2022)
Diabetic endothelial dysfunction	STZ-induced diabetic mice and HG-induced isolated rat aortas	Fenugreek 70% ethanol extract	Improves endothelial dysfunction by downregulating Arg1 expression and activity to increase NO production; simultaneously reduces Arg activity carried by serum exosomes, decreasing Arg uptake by ECs	Qiu et al. (2024)
Diabetic endothelial dysfunction	HG-induced HUVECs	Astragaloside IV-pretreated MSC-Exos	Alleviates HG-induced endothelial inflammation by delivering highly expressed miR-146a-5p to target and inhibit the TRAF6/NF-κB pathway	Chen et al. (2025b)
Diabetic wound	Diabetic rat wound model	Curcumin-loaded macrophage-derived exosomes	Promotes diabetic wound healing by activating the Nrf2/ARE pathway to inhibit oxidative stress and inflammation, enhancing HUVEC proliferation, migration, and angiogenesis	Li et al. (2023c)
Diabetic wound	HG-induced HUVECs	GExos	Reprogrammes glycolytic metabolism, restores ECs proliferation, migration, and tube formation, and promotes *in vivo* angiogenesis	Tan et al. (2024)
Diabetic wound	Diabetic wound model	Lemon exosomes loaded in GelMA-DAS hydrogel	Promotes diabetic wound healing by modulating macrophage polarization reprogramming, enhancing proliferation and migration of vascular ECs and fibroblasts	Jin et al. (2025)

DKD, diabetic kidney disease; LPS, lipopolysaccharide; TLR4, Toll-like receptor 4; DPN, diabetic peripheral neuropathy; HG, high glucose; SC, schwann cell; Stat3, Signal transducer and activator of transcription 3; GFAP, glial fibrillary acidic protein; MCP-1, Monocyte Chemoattractant Protein-1; ADSCs, Adipose-Derived Mesenchymal Stem Cells; ZEB2, Zinc Finger E-Box Binding Homeobox 2; SMAD7, SMAD, Family Member 7; BMSCs, Bone Marrow-Derived Mesenchymal Stem Cells; ELAVL1, ELAV-like RNA-binding protein 1; STZ, streptozotocin; DR, diabetic retinopathy; MSC-Exos, Mesenchymal Stem Cell-Derived Exosomes; HMGB1, High Mobility Group Box 1; Nrf2, Nuclear factor erythroid 2-related factor 2; ARE, antioxidant response element; IGF-1R, Insulin-Like Growth Factor 1 Receptor; PEDF, Pigment Epithelium-Derived Factor; ACTA1, actin, α1; PTEN, phosphatase and tensin homolog; AKT, Protein kinase B; VEGF, vascular endothelial growth factor; HUVECs, Human Umbilical Vein Endothelial Cells; EPC, endothelial progenitor cells; TRAF6, TNF, Receptor-Associated Factor 6; GExos, Ginseng-Derived Exosomes.

## 8 Future perspectives

Exosome-mediated cell-cell communication offers a novel perspective for understanding the pathogenesis of diabetic microvascular complications and shows great promise for application in diagnosis and treatment. However, despite significant advances in recent years, the field remains in its early stages, with many key scientific questions yet to be addressed and a considerable gap between current research and clinical translation.

First, the integration mechanisms of exosome-mediated communication networks in the coexistence of multiple microvascular complications remain severely underexplored. Clinically, DKD, DR, and DPN often coexist and interact within the same patient, creating a complex comorbid state (Satapathy et al., 2025). However, current studies primarily focus on local communication events in single complications, organs, or cell types. Few studies have explored how exosomes mediate cross-organ signaling integration and coordinated damage between the kidneys, retina, peripheral nerves, and the circulatory system. Future research should establish multi-organ comorbidity models and employ systems biology approaches to elucidate the cross-organ communication networks mediated by exosomes, identify shared core signaling nodes and molecular hubs, and provide a theoretical basis for developing common therapeutic strategies targeting multiple comorbidities.

Second, the clinical translation of exosomes in diagnostics faces significant challenges in standardization and validation. Although numerous candidate biomarkers have been identified in urinary and plasma exosomes, comparability and reproducibility between studies remain lacking. Future efforts should focus on large-scaleclinical validations and the development of organ-specific tracing technologies based on surface markers to improve the diagnostic specificity and etiological targeting of circulating exosomes. Exploring shared early exosomal biomarker signals across different microvascular complications also requires further in-depth research.

Third, in terms of therapeutic translation, the heterogeneity of stem cell-derived exosomes, along with challenges in large-scale production and quality control, pose significant obstacles. Factors such as the origin of mesenchymal stem cells, culture conditions, and passage number can all affect the cargo composition and bioactivity of exosomes. Future research should focus on developing stable production systems with well-defined cell line origins and controllable culture conditions to enhance the precise delivery of exosomes to microvascular units in the kidneys, retina, and peripheral nerves. Research on the mechanisms by which natural products and classic drugs regulate exosome function is still in its infancy. The specific molecular targets, signaling pathways, and cell-type specificity through which compounds like metformin, GLP-1 RAs, SGLT 2i, and astragaloside affect exosome secretion or cargo modification are unclear. Additionally, PENPs represent an emerging platform, but issues related to large-scale production, quality standards, *in vivo* pharmacokinetics, and long-term safety remain to be systematically investigated.

Finally, future research should shift from descriptive phenomena to mechanistic interventions, promoting the development of system-based therapeutic strategies for multi-disease coexistence. Building on an in-depth understanding of exosome heterogeneity and cross-organ communication networks, efforts should focus on identifying core pathogenic pathways and key exosome shared across different microvascular complications, enabling the development of interventions targeting common nodes. For instance, targeting key enzymes involved in exosome biogenesis, RNA-binding proteins that regulate pathological cargo sorting, or blocking common uptake receptors could provide a comprehensive approach. Moreover, engineered stem cell-derived exosomes could serve as multifunctional platforms, delivering anti-inflammatory, anti-apoptotic, pro-vascular stabilization, and anti-fibrotic molecules, offering multi-targeted protection for the kidneys, retina, and nerves. This multi-disease approach has the potential to overcome the current limitations, providing more effective and comprehensive treatment options for patients with diabetic microvascular comorbidities.

## Glossary

DKD: Diabetic kidney disease
DR: Diabetic Retinopathy
DPN: Diabetic Peripheral Neuropathy
EVs: Extracellular Vesicles
ESCRT: Endosomal Sorting Complex Required for Transport
miRNA: microRNA
SCs: Schwann Cells
HG: High Glucose
ILVs: Intraluminal Vesicles
MVBs: Multivesicular Bodies
VPS4: Vacuolar Protein Sorting 4
nSMase2: neutral sphingomyelinase 2
SNARE: Soluble NSF Attachment Protein Receptor
RBPs: RNA-binding proteins
LLPS: Liquid-Liquid Phase Separation
PS: Phosphatidylserine
HSPGs: Heparan Sulfate Proteoglycans
ECs: Endothelial Cells
TECs: Renal Tubular Epithelial Cells
GECs: Glomerular Endothelial Cells
RPEs: Retinal Pigment Epithelial Cells
RECs: Retinal Endothelial Cells
DRG: Dorsal Root Ganglion
SOCS: Suppressor of Cytokine Signaling
TLR4: Toll-like receptor 4
Dll4: Delta-like Ligand 4
CCL2: C-C Motif Chemokine Ligand 2
MMP2/9: Matrix Metalloproteinase 2/9
BRB: Blood-Retinal Barrier
lncRNAs: long non-coding RNAs
VEGF: Vascular Endothelial Growth Factor
RGCs: Retinal Ganglion Cells
MCs: Mesangial Cells
IGF-1R: Insulin-Like Growth Factor 1 Receptor
FOXO1: Forkhead Box Protein O1
LRG1: Leucine-rich α-2 glycoprotein 1
TRAIL: Tumor Necrosis Factor-related Apoptosis-inducing Ligand
DKK2: Dickkopf-related protein 2
WT1: Wilms Tumor Protein 1
eGFR: estimated Glomerular Filtration Rate
METTL14: Methyltransferase-like protein 14
RCN3: Reticulocalbin 3
EMT: Epithelial-to-Mesenchymal Transition
ceRNA: Competing Endogenous RNA
NVU: Neurovascular Unit
FGF9: Fibroblast Growth Factor 9
CXCL10: C-X-C Motif Chemokine Ligand 10
MyD88: Myeloid Differentiation Primary Response Protein 88
PTEN: Phosphatase and Tensin Homolog
MAC: Membrane Attack Complex
ZO1: Zonula Occludens-1
S1P1: Sphingosine-1-Phosphate Receptor 1
MSC-Exos: Mesenchymal Stem Cell-Derived Exosomes
JNK1: c-Jun N-terminal Kinase 1
AR: Aldose Reductase
GLUT1: Glucose Transporter 1
ICAM-1: Intercellular Adhesion Molecule 1
VCAM-1: Vascular Cell Adhesion Molecule 1
PDR: Proliferative Diabetic Retinopathy
SNAI1: Snail Family Transcriptional Repressor 1
MMP-X1: Matrix Metalloproteinase X1
YAP: Yes-Associated Protein
IGF1R: Insulin-like Growth Factor 1 Receptor
HECTD1: HECT Domain E3 Ubiquitin Protein Ligase 1
DNMT3A: DNA methyltransferase-3α
NUMB: NUMB Endocytic Adaptor Protein
PDPN: Painful Diabetic Peripheral Neuropathy
TRAF6: TNF Receptor-Associated Factor 6
GFAP: Glial Fibrillary Acidic Protein
MCP-1: Monocyte Chemoattractant Protein-1
AGE: Advanced Glycation End Products
FOXP1: Forkhead Box Protein P1
eNOS: Endothelial Nitric Oxide Synthase
SNAP25: Synaptosomal-Associated Protein 25
LGALS3: Galectin-3
MYH10: Myosin Heavy Chain 10
CPB2: Carboxypeptidase B2
PAK6: P21-Activated Kinase 6
Elf3: E74-Like ETS Transcription Factor 3
JUP: Junction Plakoglobin
ADSCs: Adipose-Derived Mesenchymal Stem Cells
ZEB2: Zinc Finger E-Box Binding Homeobox 2
SMAD7: SMAD Family Member 7
BMSCs: Bone Marrow-Derived Mesenchymal Stem Cells
MSCs: Mesenchymal Stem Cells
HMGB1: High Mobility Group Box 1
MAP3K1: Mitogen-Activated Protein Kinase Kinase 1
PEDF: Pigment Epithelium-Derived Factor
FOXC2: Forkhead Box Protein C2
GLP-1RAs: Glucagon-like Peptide-1 Receptor Agonists
APOC1: Apolipoprotein C1
HOXD9: Homeobox D9
SGLT2i: Sodium-Glucose Cotransporter-2 inhibitors
HUVEC: Human Umbilical Vein Endothelial Cells
ATAD3A: ATPase Family AAA Domain-Containing Protein 3A
PINK1: PTEN-Induced Kinase 1
ASIV: Astragaloside IV
EPCs: Endothelial Progenitor Cells
PENP: Plant-Derived Exosome-like Nanoparticles
GExos: Ginseng-Derived Exosomes
PFKM: Phosphofructokinase, Muscle
PGK1: Phosphoglycerate Kinase 1
ENO1: Enolase 1
